# Role of voltage-gated proton channel (Hv1) in cancer biology

**DOI:** 10.3389/fphar.2023.1175702

**Published:** 2023-04-20

**Authors:** Juan J. Alvear-Arias, Antonio Pena-Pichicoi, Christian Carrillo, Miguel Fernandez, Tania Gonzalez, Jose A. Garate, Carlos Gonzalez

**Affiliations:** ^1^ Centro Interdisciplinario de Neurociencia de Valparaíso, Universidad de Valparaíso, Valparaíso, Chile; ^2^ Millennium Nucleus in NanoBioPhysics, Universidad de Valparaíso, Valparaíso, Chile; ^3^ National Center for Minimally Invasive Surgery, La Habana, Cuba; ^4^ Facultad de Ingeniería y Tecnología, Universidad San Sebastián, Santiago, Chile; ^5^ Centro Científico y Tecnológico de Excelencia Ciencia y Vida, Santiago, Chile; ^6^ Department of Physiology and Biophysics, Miller School of Medicine, University of Miami, Miami, FL, United States

**Keywords:** Hv1, cancer, tumor, channel, proton, pH, oncology, metastasis (cancer metastasis)

## Abstract

The acid-base characteristics of tumor cells and the other elements that compose the tumor microenvironment have been topics of scientific interest in oncological research. There is much evidence confirming that pH conditions are maintained by changes in the patterns of expression of certain proton transporters. In the past decade, the voltage-gated proton channel (Hv1) has been added to this list and is increasingly being recognized as a target with onco-therapeutic potential. The Hv1 channel is key to proton extrusion for maintaining a balanced cytosolic pH. This protein-channel is expressed in a myriad of tissues and cell lineages whose functions vary from producing bioluminescence in dinoflagellates to alkalizing spermatozoa cytoplasm for reproduction, and regulating the respiratory burst for immune system response. It is no wonder that in acidic environments such as the tumor microenvironment, an exacerbated expression and function of this channel has been reported. Indeed, multiple studies have revealed a strong relationship between pH balance, cancer development, and the overexpression of the Hv1 channel, being proposed as a marker for malignancy in cancer. In this review, we present data that supports the idea that the Hv1 channel plays a significant role in cancer by maintaining pH conditions that favor the development of malignancy features in solid tumor models. With the antecedents presented in this bibliographic report, we want to strengthen the idea that the Hv1 proton channel is an excellent therapeutic strategy to counter the development of solid tumors.

## 1 Introduction

Cancer is a major public health problem worldwide because it is one of the most prevalent diseases in the world’s mortality statistics. Cancer is a disease caused by a buildup of mutations that affect vital genes responsible for cell division, cellular growth, apoptosis, protein, and DNA repair, etc., leading to an entire reprogramming of the function of a cell and/or tissue. One of the major problems of cancer is that it corresponds to a diverse disease, which affects each tissue differently, with different molecular mechanisms, and even subtle differences can be found depending on the patient’s medical history. In addition, certain types of cancer are silent until they reach very advanced stages of the disease. This can lead to significant complications when not diagnosing or treating them in a timely manner (Fisher et al., 2013; Meacham and Morrison, 2013; Hassanpour and Dehghani, 2017). The most challenging task of current medicine and science is to find a feasible and effective therapeutic pathway against cancer, identifying common characteristics that share a type of tumor within the tissue or even better, find a common feature between several types of tumor models, and aim for a therapy that includes a larger spectrum of cancers. During the past decade, it has been observed that the expression of certain ion channels is severely affected in tumoral cells compared to normal cells, favoring the development of malignant cells (Litan and Langhans, 2015). This has led to rethinking cancer as a channelopathy.

Ion channels are pore-forming transmembrane proteins present in both excitable and non-excitable cells that allow efficient ion permeability across biological membranes with high specificity. These proteins are essential for the production and transduction of electrical signals (Hille, 2001). There is a great diversity of ion channels that participate in a broad spectrum of cellular functions, where we can highlight cell communication, electrical excitability, and the maintenance of cellular homeostasis, among others (Hille, 2001). The voltage-gated proton channel (Hv1) is one such member of this large family of proteins, tasked with selective proton extrusion. Maintaining intracellular and extracellular pH within physiological values is essential for most biological mechanisms such as proliferation, mobility and apoptosis which are highly regulated by intracellular and extracellular pH. The role of the Hv1 channel in tumor biology has begun to emerge during the last decade. Currently, there has been an increasing number of studies that report that the Hv1 proton channel has a key role in proton extrusion in tumor cells, relating their overexpression in some cases to tumor malignancy (Wang et al., 2011; Wang et al., 2012; Wang et al., 2013a; Asuaje et al., 2017). For more than two decades, scientific reports have placed on the stage of tumor biology proton transporters that are key to maintain and enrich the malignant characteristics of different types of cancer (Boedtkjer and Pedersen, 2020). However, unlike the plethora of transporters that have been reported as important in cancer biology, the Hv1 protein is a proton channel that has recently been vastly explored in cancer research comparatively and its first studies date back to 2012 (Wang et al., 2012).

In this review, we discuss the evidence from the last 10 years that supports that the presence of the Hv1 channel is key to maintaining the pH conditions that characterize the tumor microenvironment which contribute to the development of cancer malignancy. Moreover, the overexpression of the Hv1 channel could serve as a predictor of incidence and cancer malignity. In addition, we discuss the potential of the Hv1 channel as a relevant therapeutic target for the development of anti-cancer drugs; thus, this protein could establish a common aim for many types of solid tumors, paving a strong strategy against tumors.

## 2 The voltage gated proton channel (Hv1) is a unique protein: structure and biophysical features

**FIGURE 1:**
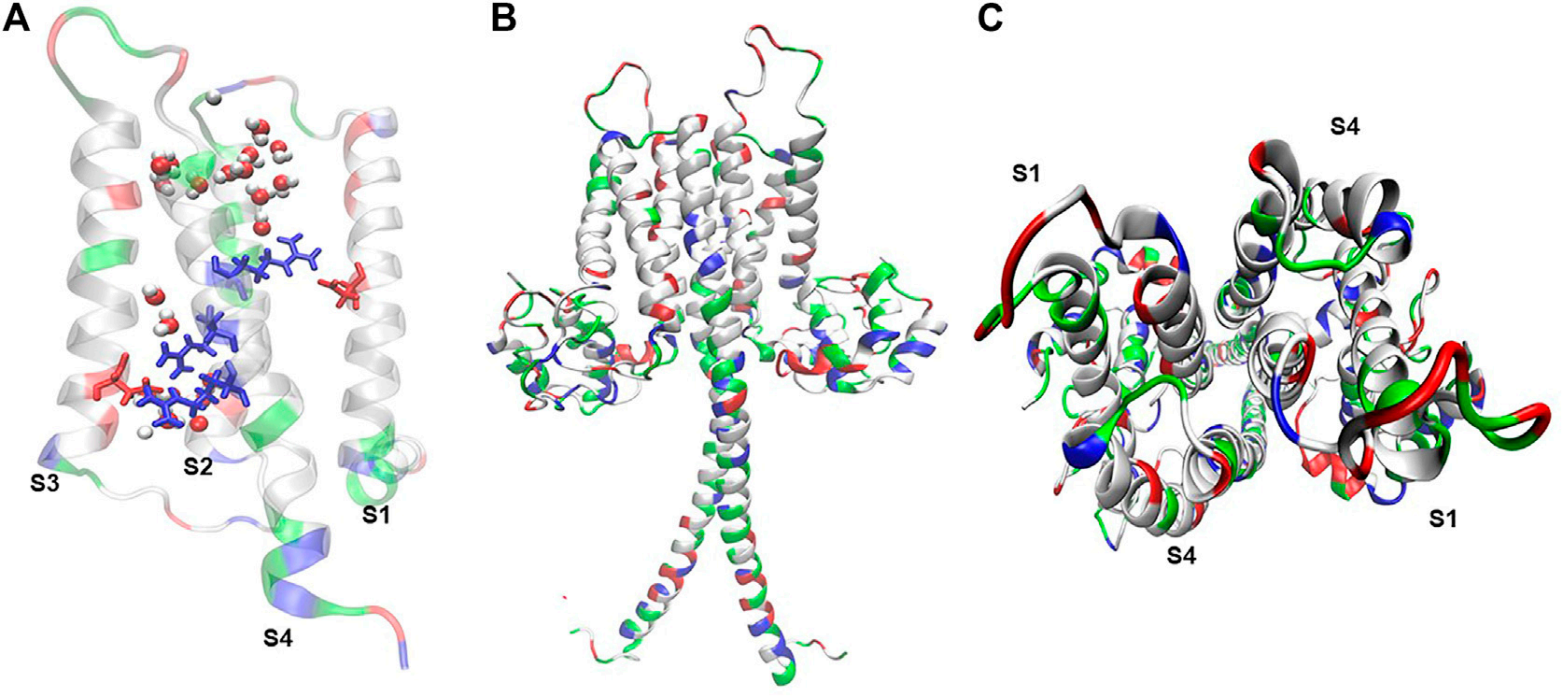
Hv1 reflects molecular arrangements that sustain its voltage-dependent conduction. **(A)** Truncated mHv1 lacking its N and C cytoplasmic terminal domains (PDB code: 3WKV, Takeshita et al., 2014) simulated in a POPC membrane bathed with a 150 mM NaCl salt solution for 0.5 ns (36141 atoms). In white: apolar residues, in green: polar residues. It is shown in blue (from up to down) three arginines related to the S4 domain, from up-to-down: Arg205 (R1), Arg 208 (R2) and Arg 2011 (R3), in red Asp112 in S1 and Asp174 in S3, and waters in VDW representation. **(B)** Full length mHv1 in dimer form, built from the best model scored of 10000 N terminal models by using Rosetta Ab initio relax application (Bradley et al., 2005), a truncated hHv1 lacking N and C terminal domains (PDB code: 5OQK, Bayrhuber et al., 2019) and the coiled-coil dimerization C terminal domain (PDB code: 3VMX, Fujiwara et al., 2012). Full length hHv1 was duplicated and positioned each monomer in front of their S1-S4 interphase following cross-linking and dimeric data (Lee et al., 2008; Mony et al., 2020) and simulated for 1 ns embedded in POPC bathed with an aqueous saline solution of 150 mM (123017 atoms). **(C)** Top view of the full length mHv1 S1-S4 dimer interface.

Hv1’s gene (*hvcn1*) was simultaneously cloned by two research groups in 2006. S. Ramsey’s group isolated the gene from human immune cells (Ramsey et al., 2006), while Okamura’s research group isolated it from mouse immune cells (Sasaki et al., 2006). This gene encodes for a ∼30 kDa proton channel, a protein whose architecture consists of four transmembrane segments (S1-S4) (Ramsey et al., 2006; Sasaki et al., 2006). This channel has unique structural and functional features. Hv1 channels are associated in homodimers (Koch et al., 2008), by the interaction of a coiled-coil motif between the C-terminus and N-terminal of each monomer (Koch et al., 2008; Tombola et al., 2008) (Figure 1). However, perfectly functional Hv1 monomers can be obtained by truncating the coiled-coil region through molecular deletions directed to the C-terminal and part of the N-terminal, which is called the ΔNΔC construct (Koch et al., 2008; Tombola et al., 2008).

To date, the Hv1 channel and its isoforms are the main voltage-gated channel that natively transports protons selectively and are within the proton transporters protein family, the most efficient to perform this task; thus, being the perfect molecular entity for maintaining complete control over pH tuning in a cellular system. In this section we will review the molecular bases of the protein.

### 2.1 Voltage sensor

The Hv1 proton channel is one of the members of the huge family of voltage-gated ion channels (VGIC). The most important section of the voltage sensor in this protein (which characterizes the members of the VGIC family) is in the S4 transmembrane helix, which contains three charged arginine residues (Arg 205, Arg 208 and Arg 211 in *Homo sapiens*, R201, R204 and R207 in *Mus musculus* and R255, R258, and R261 in *Ciona intestinalis*), which confer its voltage sensitivity (Sasaki et al., 2006; Gonzalez et al., 2010; Gonzalez et al., 2013) (Figure 1). Besides its role in voltage sensing in mHv1, R201 was shown to contribute to the channel’s outward-rectifier-like phenotype, given that its mutation to glutamine allowed the measurement of inward currents at acidic pH values (Sasaki et al., 2006).

As is typical of a voltage-gated ion channel, after membrane depolarization, the S4 helix suffers a conformational rearrangement towards the extracellular side that allows a proton-selective permeation pathway (Gonzalez et al., 2013). The motion of the charged particles of the voltage sensor produces a capacitive current that can be measured with electrophysiological techniques, these currents are known as gating currents and are a direct measurement of the conformational change of the channel during the voltage-dependent opening (Bezanilla, 2018). One of the first detections of Hv1 gating currents was obtained in 2018, during measurements of *C. intestinalis* monomeric Hv1 channels expressed in *X. laevis* oocytes membranes recorded in giant patches (Carmona et al., 2018). In dimeric Hv1 channels gating currents are difficult to resolve due to the presence of a large amount of proton current. Thus, to obtain the Hv1 gating currents Carmona et al., used a monomeric construct ΔNΔC that intrinsically possesses faster proton currents kinetics than the dimeric channel (Koch et al., 2008), with the site-directed mutation N264R that was previously observed which significantly decreases the proton currents without affecting the sensor movement (Sakata et al., 2010; Qiu et al., 2013). Thanks to this, the recording registered that ΔNΔC N264R gating currents are followed by an outward proton current and thus, the non-linear capacitive component of the recording was numerically isolated from the ON-gating current by fitting a model. Wherever this transient current satisfies all the requirements, it is called gating current (Carmona et al., 2018). Interestingly, the ΔNΔC N264R construct shows that the OFF-gating charge is apparently trapped because the OFF-gating charge only develops a small fraction of the ON-gating amplitude (1.4% of the total charge displaced during the ON) after very short depolarizations. However, the OFF-gating charge can be recovered slowly through two depolarizations separated by a variable time. This voltage protocol is known as “On-gating current recovery protocol”; to fully recover the entire gating charge takes around 87.6 ms. These observations suggest that the charge movement is more complex than an Active-Resting two state model, and the passage from the active state to the resting state is separated by a large energy barrier, which would explain why the charge is trapped. Another key point to reaffirm the sensor motion’s complexity is the evidence of two peaks revealed through the On-gating recovery protocol. This experiment gives us the information that the model has, at least, two active states. On the other hand, the two peaks revealed by the On-recovery protocol and the presence of the Cole-Moore effect also suggest three resting states to explain the kinetics of the gating currents. Furthermore, in 2021 a new construct suitable for gating currents recording was presented (Carmona et al., 2021). This new monomeric D160N construct, unlike the previous one, had the advantage that the gating charge is not trapped, and the proton macroscopic currents are almost indistinguishable, allowing a better and cleaner analysis of the gating currents (Carmona et al., 2021). These direct measurements of the sensor motion open a new and very interesting approach to address the scientific problems regarding the Hv1 channel that remain unknown because the voltage sensor, the pH sensor and the proton permeation pathway in this protein are in the same structural domain, as we will later discuss.

Besides S4, the external end of the S1 helix exhibited a weak voltage-dependence, while a strong voltage-dependence was reported at the internal end of the S1 helix (Mony et al., 2015). Voltage-patch clamp fluorometry measurements tracking the S1 kinetics are consistent with the motion of S1 coupled to the opening of Hv1 (Mony et al., 2015). The S2 helix has been reported to contribute to the voltage response of Hv1, as the mutation hHv1 D153C exhibited a displacement to the left of their GV curves, suggesting this residue is relevant to the gating process (Tombola et al., 2010). By voltage-clamp fluorometry, it was shown that voltage sensing and pore opening are concerted events, where voltage-sensing precedes opening (Tombola et al., 2010; Mony et al., 2015).

### 2.2 pH sensor

In addition to proton selectivity, the Hv1 currents show several interesting electrophysiological hallmarks that allow researchers to differentiate them from other ion channels. The macroscopic currents of the Hv1 channel are highly dependent on the pH difference between the extracellular and intracellular side of the membrane (ΔpH = pH_extracellular_ (pHo)—pH_intracellular_ (pHi) (Cherny et al., 1995). As a result of this, the activation curves of the macroscopic currents shift to negative voltages at positive ΔpH, indicating that channels require less electrical energy to achieve the open state (Cherny et al., 1995; Alvear-Arias et al., 2022). Interestingly, the G-V curves are only affected by the ΔpH and not the absolute pH. This means that a ΔpH = 2 curve made by pH_i_ = 5.5//pH_o_ = 7.5, has the same parameters of half-activation voltage (V_0.5_) and zδ as a ΔpH = 2 curve made by pH_i_ = 4.0//pH_o_ = 6.0 curve (Schladt and Berger, 2020). However, the channel kinetics are sensitive to the absolute pH values, i.e., it depends on the internal and external pH values instead of ΔpH, showing a more sensitive response to the intracellular acidification (Schladt and Berger, 2020). Also, the unitary conductance of Hv1 channels is sensitive to absolute pH, precisely to the intracellular pH. It has been estimated at 38 fS both at ΔpH 0 with pH_i_ = 6.5 and pH_o_ = 6.5 and ΔpH 1 with pH_i_ = 6.5 and pH_o_ = 7.5, suggesting that changes in extracellular pH bring no effect over unitary conductance, while at ΔpH 2 with pH_i_ = 5.5 and pH_o_ = 6.5 it was estimated approximately 140 fS, which, together with the previous suggestion, strongly propose that the unitary Hv1 conductance is sensitive to absolute intracellular pH (Cherny et al., 2003). These quantities represent nearly one hundredth to one-thousandth of microscopical conductance observed in most of the ion channels known to date (Cherny et al., 2003). Recently, it has been suggested by direct measurement of the gating charge displacement that the voltage sensor is responsible for ΔpH dependence in the Hv1 channels (Carmona et al., 2021). These studies were carried out in the monomeric D160N construct, a site-directed mutation to the selectivity filter which by replacing it with asparagine, severely impairs proton conduction, allowing it to detect and measure clean gating currents by strongly reducing the proton currents. Surprisingly, the voltage sensor rearrangement upon membrane depolarization is strongly modulated by ΔpH changes, observing a leftward shift in the ON-gating charge versus voltage curves (Q-V) as the ΔpH is higher on the experimental conditions (Carmona et al., 2021). Equally as the G-V curves, the Q-V relationship is modulated only by the ΔpH and not by the absolute pH, however the activation kinetics and deactivation kinetics are modulated by absolute pH values, equally as observed in the macroscopic current kinetics (Carmona et al., 2021). This evidence shows that the energy stored due to the electrochemical gradient caused by the protons on the extra- and intracellular side is used in some measure to activate the voltage sensor. In fact, 60% of the stored energy related to the ΔpH is coupled to the voltage sensor. It is thanks to this that the expression of the total free energy needed by the system to describe this coupling has the form,

$$∆G=G_{ex}-G_{in}={∆G}^{0}0-2.3eRT∆pH-zFV$$

On the other hand, this coupling could be an important mechanism for tumor cells to control their homeostasis and thus regulate the waste that originates inside them due to their metabolism.

This data is interesting because it suggests that the modulation of the voltage sensor is involved in the ΔpH sensitivity of the channel. However, the molecular determinants of the internal and external pH sensitivity are still unknown, and this last one has been the piece of the Hv1 puzzle that has kept many research groups busy for years.

### 2.3 Permeation pathway and selectivity filter

As we presented earlier, the monomeric ΔNΔC construct is perfectly functional (Koch et al., 2008; Tombola et al., 2008). This is quite interesting because it is the absolute proof that each monomer possesses its own voltage sensor, pH sensor and proton permeation pathway, and that means that all these three critical functional domains are contained in the same structural region. Thus, when studying one of these functions and its molecular determinants, you are indirectly studying the three of them; this makes the Hv1 channel a particularly challenging protein.

The permeation mechanism of the Hv1 channel is still controversial to date. The controversy emerges due to the lack of an open structure, in fact, the best template of a crystal structure of the protein is Okamura’s laboratory chimera (Takeshita et al., 2014). This chimeric channel, built up mainly from parts taken from mHv1, *Ci*Hv1, *C. intestinalis* voltage-gated phosphatase (C*i*Vsp), was crystallized in the presence of zinc ion to stabilize the protein. For this reason, the crystal state is unclear, and this state was termed as intermediate resting (IR) (Takeshita et al., 2014). Therefore, researchers need to build models to generate an open structure. Moreover, at present there is an open model of the channel that was generated utilizing molecular dynamics, which was opened with voltage using microsecond simulation times.

There are two different hypotheses for the Hv1 proton permeation proposed to date. The first one suggests that a continuous of water, like a wire of water molecules across the entire permeation pathway from the intracellular to extracellular bulk, while the protein is in its open conformation (Ramsey et al., 2010). In this model, protons move towards the wire into the hydrogen bonds railways like the *Grotthuss* mechanism (de Grotthuss, 2006), where the protonation of residues is not needed.

**FIGURE 2:**
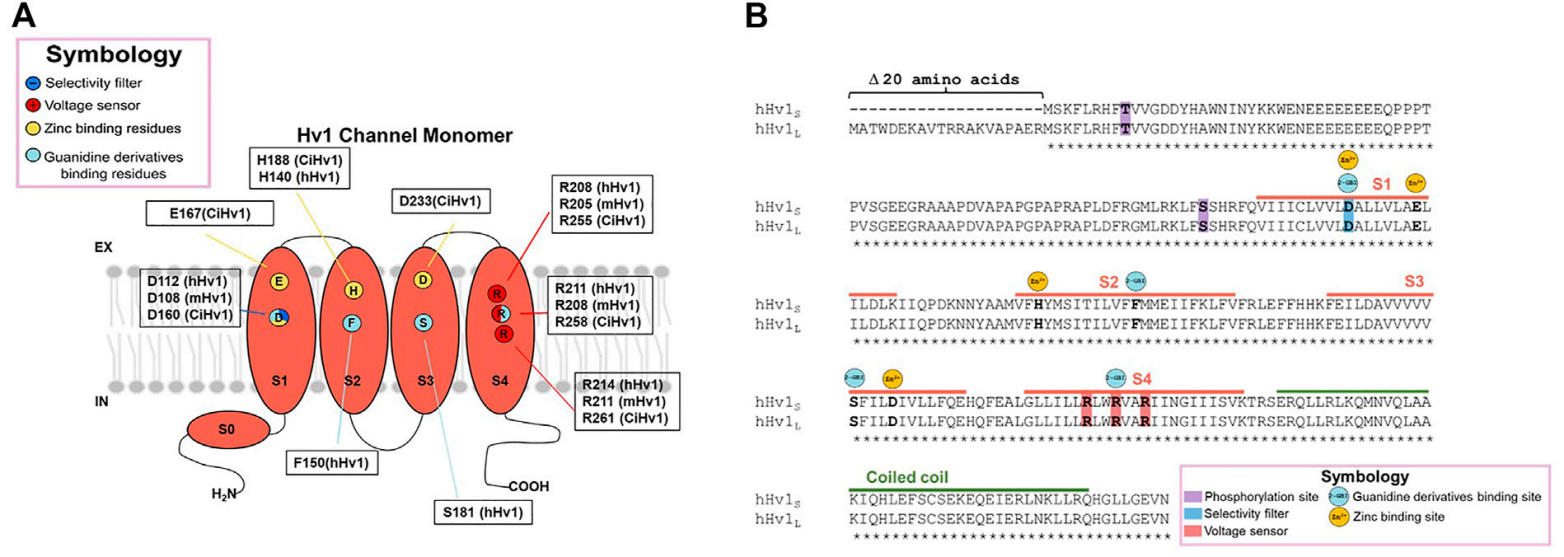
Structure of the Hv1 channel and molecular determinants of Zn^2+^ and Guanidine derivatives binding sites. The molecular determinants in the interaction of the protein with the classical inhibitors zinc ion and guanidine derivatives are pointed in **(A)** the monomeric channel cartoon, indicating in what species were discovered the interaction with the chemical compounds and, **(B)** in the sequence alignment between long and short Hv1 isoforms.

The second hypothesis states that proton permeation occurs thanks to the protonation of the selectivity filter, an aspartic acid (negative charged amino acid, Asp 112 for human Hv1, D108 for mHv1) located in the transmembrane segment S1 (Musset and DeCoursey, 2012) (Figure 2A). Moreover, the water wire that is established from bulk to bulk is interrupted by salt bridges between the selectivity filter and the second and third arginine of the voltage sensor, while the channel is in its open conformation. At this point the aspartic acid is protonated to complete the H-bond chain during the open state allowing proton selectivity without losing efficiency in transport (Sakata et al., 2010; Berger and Isacoff, 2011; Kulleperuma et al., 2013; Morgan et al., 2013; Dudev et al., 2015). Some studies in molecular dynamics (MD) suggest that there is no dry or a free water zone in the Hv1 channel (Gianti et al., 2016). Therefore, the starting point to finally understand the permeation mechanisms of Hv1 should be to thoroughly study the behavior of water inside the channel. As discussed in the previous section, pH variations are a determinant factor in the channel’s functioning. Undoubtedly, the connection between the transport of protons and water molecules cannot go unnoticed and understanding these mechanisms could not only contribute to the understanding of the structure-function relationship of the channel, but also, as we will further see, the proton permeation establishes a bridge to the processes in which the Hv1 channel is directly involved in cancer development.

### 2.4 Proton depletion in Hv1 channels

One of the distinctive features of a proton current that could be noticed during an electrophysiological recording is proton depletion (De-la-Rosa et al., 2016). In an electrophysiological recording, this phenomenon looks like a reduction in the current amplitude in time, which is more dramatic at higher depolarizations. At a glance, the proton depletions could be confused with a fast inactivation process such as a shaker potassium channel, sodium channel and many others. However, the phenomenon of fast inactivation is a voltage-dependent state related to the motion of a cytoplasmic region (known as the inactivating particle) that could be part of the protein or even an extrinsic particle, that moves towards the pore and physically obtrudes the ion fluxes (Goldin, 2003). Due to the abovementioned, the inactivation in an electrophysiological recording induces a dramatic reduction in the ON current amplitude (Armstrong and Hollingworth, 2018). The genesis of the proton depletion phenomenon does not correspond to a conformational change or involves the intervention of a protein motive, as far as we know, but emerges due to the very efficient proton transport through the proton channels (De-la-Rosa et al., 2016). The proton concentration in a physiological context is lower than the concentration of other membrane permeable ions such as sodium and potassium. In a membrane in resting conditions, Na+ and K+ are in millimolar concentrations, while H+ in a physiological pH, are in nanomolar concentrations. The efficient transport towards the extracellular media causes a drastic reduction of the protons available in the vicinity of the proton channels. Thus, the concentration ratio between the intracellular and extracellular proton concentration tends rapidly towards equilibrium causing a reduction in the electromotive force for protons, inducing a drop in the current amplitude which could be observable in a millisecond timescale. This is quite interesting because this electrophysiological feature is an undeniable sign of how efficient a proton channel can be when regulating drastic changes in intracellular pH, and for this very reason, the Hv1 channel has been found in tissues where the intracellular pH control must be perfectly tuned and moreover, its overexpression could be critical for tumoral development and malignancy behavior, as we will later present.

## 3 Pharmacological properties of Hv1 channel

Although up to today now several drugs and new molecules that modulate the function of Hv1 channels have been discovered, there are two principal species studied among them, which act as inhibitors of Hv1 channel: Zinc ion and 2-guanidine benzimidazole (2-GBI) derivatives.

### 3.1 Unspecific blocker, Zinc ion

For many years, zinc and other divalent cations were used as the main proton current inhibitors (Thomas and Meech, 1982). The divalent metal is a reversible blocker that, when added extracellularly at micromolar concentrations (μM), decreases the speed of channel opening and shifts the activation curve towards more positive voltages, indicating that the binding to the protein stabilizes a non-conductive state (Thomas and Meech, 1982; Morgan et al., 2007; Qiu et al., 2016). The dissociation constant, K_d_, for the blockade of human lymphocyte Hv1 currents by zinc ion was voltage-dependent, observing that K_d_ was equal to 8 nM at +40 mV, 36 nM at +70 mV and 0.2 μM at +100 mV (Gordienko et al., 1996). Regarding the inhibition mechanism, for the binding site at resting closed state, the contribution of the extracellular end and the middle of S1 and S3, by the residues D160, E167, D233 and H188, was proposed as the second binding site, perturbing the outward displacement of S4 at membrane depolarization, while H188, D233 and E167 contribute to binding the zinc ion to the first binding site, which prevents channel opening (Figure 2) (Qiu et al., 2016). It has also been observed that Zn^2+^ inhibition is dependent on ΔpH, since the current inhibition in the presence of the divalent cations becomes more drastic as the difference in extracellular and intracellular pH increases (Cherny and DeCoursey, 1999).

### 3.2 Specific blocker, guanidine derivatives

Several molecules derived from guanidine have been described to elicit a decrease in proton currents. Among them, the permeable membrane 5-chloro-2-guanidinobenzoate imidazole (ClGBI) produces the sharpest and most effective inhibition of currents (Hong et al., 2014). Measurements at +120 mV (and pHi = pHo = 6.0) were blocked by additive concentrations of ClGBI, evidencing a K_d_ for the human Hv1 of 38.3 ± 0.7 μM. The K_d_ was voltage-dependent, exhibiting a decrease in its value the more depolarized the channel, with the K_d_ ranging from nearly 100 μM to 30 μM from 20 mV to 140 mV, respectively (Hong et al., 2013). In contrast, in the inhibition by guanidine derivatives, the blocking mechanism has been proposed to occur in the intracellular side during the open state, with a binding site located between the permeation pathway and the cytoplasm, specifically binding to the residues D112 in S1, F150 in S2, S181 in S3 and R211 in S4 in hHv1 (Hong et al., 2013; 2014) **(** Figure 2B **)**.

### 3.3 Other blockers of Hv1 channel

The *de novo* designed peptide Corza C6 was made by the phage display technique, using an inhibitor cystine knot scaffold to build nearly 1 million novel peptides. C6 selectively inhibits Hv1 over a broad nanomolar concentration, 0.01–10000 nM, with an inhibition constant, measured at 40 mV of 30.9 ± 3.4 nM (Zhao et al., 2018). Hanatoxin (HaTx), a compound found natively in tarantula venom, was found to inhibit Hv1 at depolarized potentials (30 and 60 mV) by the application of 4 μM of HaTx (Alabi et al., 2007). The Antitumor analgesic peptide (AGAP) found in the scorpion toxin of *Buthus martensii* was observed to strongly inhibit Hv1 currents. It was found that the structural arrangement involved in stabilization of Zn2+ ions is closely related to AGAP stabilization in Hv1 channels. At +30 mV, it detected an IC_50_ value of 2.5 +- 0.4 μM, when sampled from a range of concentrations of 0.001 to near 10 μM. Over the structural arrangements of ClGBI, new inhibitors have been developed, such as those proposed by the group of F. Tombola known as “Hv1 Inhibitors Flexibles” (HIFs) (Zhao et al., 2021). These compounds act over a range of concentrations from 1 to 100 μM, with an IC_50_ of 13.3 ± 1.0 µM. Recently, the selective synthetic inhibitor YHV98-4 was discovered to selectively inhibit Hv1 currents over a range from 0.1 to 100 μM, with an IC_50_ reported of 1 μM (Zhang et al., 2022). Therapeutically, it can reduce the alkalinization of Hv1 expressing neurons and ROS production in chronic pain, which in turn reduces pro-inflammatory chemokines liberation (Zhang et al., 2022).

Some drugs used to treat certain diseases have been shown to inhibit Hv1 currents. A few reports have suggested that antipsychotics like chlorpromazine, haloperidol, clozapine, olanzapine and risperidone could be Hv1 inhibitors (Shin and Song, 2014; Shin et al., 2015). It has been shown that a decrease in the proton current amplitude in mouse microglial BV2 cells occurs in response to micromolar concentrations of these blockers of dopamine receptors in the extracellular solution (Shin and Song, 2014; Shin et al., 2015). Another report has shown that antidepressant drugs such as imipramine considerably reduces the proton currents in mouse microglial BV2 cells (Song et al., 2012). However, in all the cases mentioned above, further studies must be performed in order to identify the specific mechanism of the inhibition and the molecular determinants responsible for the binding of these compounds to the channel.

### 3.4 Activators of Hv1 channel

There is little evidence regarding enhancement of proton currents by pharmacological agents. Few activators of Hv1 similar to unsaturated fatty acids have been reported, the arachidonic acid in most detail, which induces an increase in the intensity of currents when applied in a concentration interval of 10–100 μM (Kawanabe and Okamura, 2016). All pharmacological modulators of the function of Hv1 are gathered in Table 1.

**TABLE 1:** Pharmacological modulators of Hv1 channel activity and its physiological relationship.

Compound	Type	Concentration interval	IC_50_	Effect on channel	Physiological effect	References
**Zn** ^**2+**^	Cation	0.001–2 mM	2 μM	Inhibitor (extracellular)	Attenuates the immunosuppressive function of MDSC	Cherny and DeCoursey (1999), Ramsey et al. (2006), Alvear-Arias et al. (2022)
**Cd** ^**2+**^	Cation	1-100 μM	-	Inhibitor (extracellular)	-	DeCoursey (2018), Mahaut-Smith (1989), Sasaki et al. (2006), Thomas and Meech (1982)
**ClGBI**	Chaotropic agent	0.01—2 mM	38 μM	Inhibitor (intracellular)	Anti-proliferative of cancer cells; Attenuates the immunosuppressive function of MDSC	Hong et al. (2013), Ventura et al. (2020), Alvear-Arias et al. (2022)
**YHV98-4**	Synthetic compound	0.1–100 μM	1 μM	inhibitor	Analgesic and anti-inflammatory in chronic pain	Zhang et al. (2022)
**HIFs**	Synthetic compound	1–100 μM	13.3 ± 1.0 µM	Inhibitor (intracellular)	-	Zhao et al. (2021)
**Corza C6**	*De novo* designed peptide	0.01–10000 nM	30.9 ± 3.4 nM	inhibitor	Suppression of the acrosome reaction in human spermatozoa	Zhao et al. (2018)
**Hanatoxin (HaTx)**	Tarantula venom	4 μM	-	inhibitor	-	Alabi et al. (2007)
**Antitumor analgesic peptide (AGAP)**	Scorpion toxin	0.001 to near 10 μM	2.5 ± 0.4 μM	inhibitor	-	Tang et al. (2020)
**Arachidonic Acid**	Unsaturated- fatty acid	10–100 μM	50 μM	activator	Intensifies respiratory burst on WBC	Kawanabe and Okamura (2016)

Favorably, most inhibitors of Hv1 operate in small concentrations, accompanied by a high selectivity; hence, these pharmacological properties can be useful to study the role of Hv1 in different cell types, enabling the elucidation of physiological aspects regarding determined tissues without intervention of other signals related to concentrations of drugs that could trigger these. In the following sections, both the role of Hv1 in physiology and pathophysiology are mentioned in detail, especially in cancer.

### 3.5 Post-translational features and isoform of Hv1

Although the macroscopic activation of Hv1 channels is dependent on the pH difference between both sides of the membrane, an interesting study revealed that the human sperm possesses a truncated Hv1, named Hv1Sper, which emerges by a cleavage in the N-terminal (Hv1-Δ68) of full-length mHv1, by a serine protease (Berger et al., 2017). The truncation confers activation sensitivity to absolute pH (Berger et al., 2017). These cleaved mHv1 channels do form heterodimers together with full length mHv1 (Berger et al., 2017). This data helps us understand that the N-terminus is involved in pH-sensitivity, but it does not compromise the ΔpH sensitivity of the channel. Studies carried out with the perforated patch clamp technique in primary cultures of immune system cells have reported an effect called “enhanced gating mode,” which is a phenomenon that occurs after stimulation with phorbol 12-meristate-13-acetate (PMA). PMA addition to the extracellular side of cells promotes a shift of activation curves of Hv1 channel currents to negative voltages and faster opening kinetics (Bánfi et al., 1999; DeCoursey et al., 2000; 2001). Mechanistically, it operates by activating the phospholipase C (PLC) enzyme which utterly leads to the activation of protein kinase C (PKC), which can selectively phosphorylate Threonine 29 in the N-terminal of hHv1 (Figure 2B), leading to the enhancement of its gating (Musset et al., 2010). It has been shown that this activation is also stimulated and sustained by PKC inducing the channel’s phosphorylation (Morgan et al., 2007). Two isoforms of the Hv1 channel found to date differ in the length of the beginning of the N-terminal domain (long and short isoforms have been found; Long Hv1: NP_001035196.1 and Short Hv1: NP_001243342.1), with the other big part of the protein absolutely conserved (Capasso et al., 2010; Hondares et al., 2014; Ventura et al., 2020). As can be seen in Figure 2B, the binding sites for currently known inhibitors (such as Zinc ion and guanidine derivatives) are all present in this very highly conserved portion among both isoforms. Given that, we expect the inhibitors to bind and attenuate the function of both long and short Hv1. This is quite encouraging when proposing and developing new oncology drugs since, regardless of the isoform that a tumor may present, the drugs would have inhibitory activity. Nevertheless, when considering *in vivo*, there are multiple other factors that can modulate the inhibitory activity, therefore it could be important to expand the *in vitro* pharmacological investigation to *in vivo* models. With respect to the design of Hv1 inhibitors, and although there are approaches that selectively target certain portions of the amino acid sequence of Hv1 channels, such as the phage-display technique (Zhao et al., 2018), much work remains to be done in the field of inhibitor design in Hv1 channels, since many drugs that have been developed have been mainly based on the pharmacophore GBI. Moreover, as we present in Table 1, there are several compounds that have been shown to have inhibitory effects on the channel, however the molecular mechanism by which these exert their action is still obscure.

All this evidence related to the biophysical features of the Hv1 channel described above helps us understand why this protein has a fundamental role when it comes to regulating the pH of cellular systems in such a rigorous and perfectly tuned manner.

## 4 Tissue expression and physiological functions of the Hv1 channel

As discussed in the previous section, the biophysical properties of the Hv1 proton channel are tremendously convenient when it comes to pH control. In fact, in terms of efficiency and speed, the proton channel is the ideal protein for the task. There are many acid extruders that in different tissues collaborate with the transport of protons across membranes or with the pH regulation via buffers. Examples of these are Na + -H+ exchanger (NHE) family, H + -ATPase, monocarboxylate transporters (MCT) family, Otopetrin channel (OTOP) family, Na + -dependent Cl-/HCO3- exchanger, among others. However, most of these correspond to active (pumps) or secondary (ion transporters) transport, with slower rate compared to the voltage-gated proton channel, Hv1, that moves protons in favor of their electrochemical gradient. The channel’s expression is not only able to support a fast and efficient proton efflux during biological processes that acutely acidify the cytosol, but also, and depending on the tissue, the Hv1 proton transport is functionally coupled to specialized cellular functions, such as those that allow protection against bacteria, i.e., the respiratory burst, to mention, by highly regulated molecular mechanisms, extending the importance of its expression in healthy cells. The Hv1 channel expression is reported in a broad diversity of cell types that form part of the respiratory system, the reproductive system, the endocrine system and the nervous system. However, the most physiologically elevated expression of Hv1 is found among the members of the immune system. All these functional systems are particularly important in the biology of mammals, such as mice and humans. In the following section, we will briefly go over the physiological tasks performed by the Hv1 channel.

### 4.1 The respiratory system

In the airway epithelium, the Hv1 channel regulates the extracellular pH in the airway surface liquid (DeCoursey, 1991). Interestingly, the only naturally occurring mutation of Hv1 has been reported in human airway epithelia, where the M91T substitution confer insensitivity to zinc to lessen acid secretion from primary cultures of the trachea from humans at mucosal pH 7.5 while manifesting inhibition at mucosal pH 8.0, and a shift of its I-V currents to the right in comparison to the WT, suggesting that the mutation influences the activation of Hv1 in human airway epithelium. Although the amino acid M91 is not highly preserved among species, it is in *Equus caballus*, while the T91 amino acid appears to be preserved in *Macaca mulatta* and *Bos taurus* (Lovannisci et al., 2010).

### 4.2 The Reproductive system

The intracellular pH of sperm is crucial for controlling activation and further flagellar motility. This is because the spermatozoa are stored inactive in the caudal part of the epididymis and this inactivation is caused by intracellular acidification and an acid extracellular environment. To initiate the process of sperm activation and flagellar motility cytosolic alkalinization is required (Carr and Acott, 1984; Carr and Acotf, 1989; Hamamah and Gatti, 1998). In 2010, it was shown that human sperm possesses the Hv1 channel expressed in the main part of the sperm flagellum (Lishko et al., 2010) with the channel being the main actor in motility. Later in 2017, a shorter variant that lacks 60 amino acid residues from its N-terminal with a molecular weight of ∼25 kDa, the Hv1Sper, was discovered (Berger et al., 2017). This variant is given by a post-translationally proteolytic cleavage led by a serine protease at position R68 or A69. The opening kinetics of Hv1Sper was shown to be faster the higher the pH_i_ and pH_o_, while in the established Hv1 it is the opposite: the lower the pH_i_ and pH_o_, the faster the channel opens (Berger et al., 2017). It was shown that the expression of this truncated form is enriched in highly motile sperm, compared to motile and immotile sperm, which suggests that this shorter variant might be boosting sperm motility (Berger et al., 2017). With these studies, the proton channel is gaining attention in male fertility studies.

### 4.3 The Endocrine system

Recent studies have shown that Hv1 is expressed functionally in the pancreas and has a significant role in the regulation of endocrine function (Zhao et al., 2015; Pang et al., 2020). The pancreas is an organ with mixed behavior, as it has both exocrine and endocrine secretion. The endocrine portion is responsible for the secretion of insulin and amylin through cell clusters called Langerhans Islets. It has been shown that human and mouse islets express Hv1 channel (Zhao et al., 2015), observing that the proton channel is located preferentially in insulin-containing vesicles. Furthermore, the genetic silencing of the *hvcn1* gene in isolated islets and in INS-1 rat insulinoma cell line, showed a decrease in insulin secretion. The control of the intracellular pH in the β-cells and its proper regulation is essential in the secretory machinery of insulin, which is the pivotal hormone in glucose homeostasis in mammals. It has been shown that Hv1 KO mice possess an impaired metabolism phenotype characterized by hyperglycemia and glucose intolerance due to a reduction in insulin secretion by the beta cells (Pang et al., 2020; Pang et al., 2021). PMA stimulation (5 µM) of islet β-cells evoked a secretion of 2.5 ng of insulin per islet, while for the KO hvcn1 β-cells PMA induced a secretion 2.5-fold lower insulin per islet, 1 ng of insulin per islet. These works are tremendously valuable as they demonstrate that the Hv1 channel is an important pH-tuner during insulin secretion, glucose homeostasis and energetic metabolism.

In the past decade, studies showing that deregulation of Hv1 expression leads to disease have increased, also expanding the diversification of functions in different tissues. This has given an interesting twist to the protein as more than a simple regulator of cellular pH, it has become a protein of therapeutic interest in translational science related to the fields of neuroscience, immunology, and cancer, as we will describe below.

### 4.4 The Nervous system

Hv1 channel is essential for the function of microglia in the nervous system. It does support ROS production and intracellular pH homeostasis (Li et al., 2021; Peng et al., 2021). An Hv1 WT mice spinal cord injury contusion model presented an upregulation of the mRNA levels of mHv1 in microglia, while the Hv1 KO counterpart exhibited decreased tissue acidosis, accompanied by diminished ROS levels (Li et al., 2021). Interestingly, the KO model showed a decreased expression of a set of neuroimmune and cytokine genes, resulting in an improved recovery of the wound (Li et al., 2021). In another mice contusion model, of spinal nerve transection, an increase of electrophysiologically functional Hv1 in spinal glia was detected (Peng et al., 2021). In the same contusion model, but with Hv1 deleted by KO, a reduction in ROS generation, a diminished astrocyte activation, downregulation of IFN-γ in spinal astrocytes and lower pain hypersensitivity posterior to the injury was observed (Peng et al., 2021). The *in vitro* deletion of Hv1 exhibited an enhanced cell migration of microglia and macrophages, while the *in vivo* neutralization of Hv1, via antibody, promoted microglial migration and the clearance of myelin debris (Wang et al., 2021). The role of Hv1 in the phagocytosis of myelin was studied by generating a Hv1 KO 293T culture cell model and an Hv1 overexpressed 293T cell system, concluding that neither deletion nor overexpression of HVCN1 in 293T cells were affecting myelin phagocytosis; in contrast, Hv1 neutralization with antibodies *in vivo* induced an intensification of myelin debris clearance in the brain (Wang et al., 2021). As well, Hv1 in microglia was assigned as a marker of ischemic stroke (Eder and DeCoursey, 2001). Hv1 channel was recently found in peripheral sensory neurons in dorsal root ganglia (DRG) from humans and rodents (Zhang et al., 2022). The expression of the channel is upregulated during neuroinflammation, where the proton channel promotes the intracellular alkalinization and ROS production that, in DRG, is related with pain. Thus, the overexpression of the Hv1 channel is enhancing nociception and aggravating inflammation (Zhang et al., 2022). The relationship between the Hv1 channel and ROS production seems to be a feature of inflammatory scenarios as we will see in the following section.

### 4.5 The Immune system

Hv1 seems to be intimately related to the immune system. Functional expression of Hv1 has been reported in monocytes and microglia, T and B lymphocytes, in granulocytes (neutrophils, eosinophils, and basophils), and dendritic cells (Nanda et al., 1994; Schilling et al., 2002; Musset et al., 2008; Morgan et al., 2009; Zhu et al., 2013; Hondares et al., 2014; Asuaje et al., 2017; 2018). Here, the role of Hv1 in pro-inflammatory myeloid derived cells is widely described and is related to enabling the sustained production of reactive oxygen species (ROS) via NADPH oxidase 2 (NOX2) during respiratory burst. The oxidation reaction of NADPH produces electrons which are transported through the transmembrane subunit gp91 to the extracellular side, where the enzymatic complex reduces O2 to O2- from which other ROS species can appear (Kusmartsev and Gabrilovich, 2006). NADPH oxidation produces NADP+, H+ and two molecules of O2-, thus, each cycle of enzymatic activity causes the acidification of the cytosol (Henderson et al., 1988). It has been reported that the optimal activity of this enzymatic complex is close to pH_i_ 7 (Babior et al., 1973; Henderson et al., 1988), and that when reaching pH_i_ 5.5, the enzymatic activity of NOX2 is irreversibly inhibited (Morgan et al., 2005). On the other hand, the accumulation of H+ produces a local difference in electrical potential around the enzyme. The activity of this enzyme is independent from electrical potential in the range of −100 mV–0 mV; however, if this potential range is exceeded, the activity of the enzymatic complex begins to decrease until disappearing at voltages close to +200 mV (Decoursey, 2003). Theoretical considerations suggest the enzymatic activity without a compensatory mechanism of membrane potential, would generate a depolarization close to +200 mV (Decoursey, 2003). For this system to be functional and so it can operate in a sustained manner, the presence of the Hv1 channel is crucial as it is the molecular entity capable of maintaining both the membrane potential (E_m_) and the cytosolic pH at levels that allow the proper functioning of the enzymatic complex.

Opposite to proinflammatory immune cells, myeloid-derived suppressor cells (MDSC) (Alvear-Arias et al., 2022), which are members of the immune system derived from myelopoiesis responsible for controlling the end of the inflammation processes using this functional coupling between Hv1 channel and NOX2 complex to promote its anti-inflammatory activity, releasing extracellular ROS to inhibit T cell activation and proliferation (Alvear-Arias et al., 2022). This heterogeneous population of suppressor cells is an important part of the Tumor Microenvironment (TME) in solid cancer, seeming to be recruited directly from bone marrow to the location of solid tumors (Panyi et al., 2014; Tesi, 2019; Jin and Jin, 2020; Alvear-Arias et al., 2022). In myeloid precursors isolated from bone marrow of mice, the expression kinetics of Hv1 is observed to increase along the differentiation of the cells (Alvear-Arias et al., 2022). It has been observed that, through several factors secreted by tumor cells, MDSC are recruited and differentiated in the tumor microenvironment so that they exert their suppressive activity on T lymphocytes. Mouse MDSCs can be described by their expression of the surface markers Gr-1 (Ly6C/Ly6G) and CD11b and then further classified into two main phenotypes: monocytic (MO)- MDSCs (Gr-1^low/int^CD11b^+^Ly6C^hi^Ly6G^−^) and polymorphonuclear/granulocytic (PMN)-MDSCs (Gr-1^hi^CD11b^+^Ly6C^low^Ly6G^+^), both able to suppress antigen-specific response of T cells (Movahedi et al., 2008; Youn et al., 2008; Peranzoni et al., 2010). When trying to make the same characterization in humans, it gets trickier as humans do not express the Gr-1 marker *per se*, so the hMo-MDSC are described as HLA-DR^-^ CD11b^+^ CD14^+^ CD15^−^ CD33^high^ and hPMN-MDSC as HLA-DR^-^ CD11b^+^ CD14^−^ CD15^+^ CD33^Mid^ (Cassetta et al., 2019). The immunosuppressive role of MDSC in the tumor microenvironment has attracted the interest of many researchers as one of the multiple therapeutic targets in immunotherapy. At the same time, the Hv1 channel was recently shown to be an important element in the production of ROS during MDSC immunosuppression (Alvear-Arias et al., 2022), a cellular mechanism triggered by cancer, which weakens the immune anti-tumoral system, attempting to elevate its survival and proliferation. Then, Hv1 is implicated in cancer progress, as this protein is present in some of the cellular elements that compose the malignant microenvironment and sustain the growth of malignant cells. Thus, this channel could be a possible molecular target utilized for cancer therapies, as we will see in detail in the next section.

## 5 Hv1 channel in cancer biology: pH control as one of the governing axis of cancer

Cancer is one of the principal diseases of the modern world, with reports owing to both high incidence and prevalence worldwide (According to the Global Cancer Observatory, WHO, https://gco.iarc.fr/, last visited in January 2023). Even when there are plenty of investigations of the disease, nowadays, it is still a task to determine the mechanisms by which it exerts its malignancy. Several efforts have resulted in the elucidation of several characteristics regarding how cancer cells associate together to form a tumor. All share a common issue: they promote the acidification of their environment. These cells present an increased glucose uptake and fermentation to lactate, known as the Warburg Effect. When cells suffer the Warburg effect, they tend to produce a substantial quantity of protons in their cytosols, but they exhibit minor alkaline pH_i_ levels, compared to counterpart cells in homeostatic physiological conditions (Helmlinger et al., 1997). This pH configuration is a central axis in tumor biology, principally because it allows the survival of cancer cells to cytosolic acid stress and promotes their malignancy. It has been discovered that the existence of several proton transporters and exchangers are tightly associated with the tumor microenvironment, which allows for this pH regulation. Even though the central paradigm of this review is Hv1, it doesn’t mean that it is the only proton extruder that can be present in tumors: Some other examples of proton extruders and their relationship with cancer biology are: i) Na^+^/H^+^ exchanger 1 (NHE1). NHE1 is a secondary active transporter that internalizes a sodium ion while extruding a proton. Overexpression of the NHE1 has been reported in certain types of cancer cells, such as colorectal cancer and breast cancer cells where it contributes to proton efflux and regulates the cytosolic pH of the tumor cells (Lopez-Charcas et al., 2022), the pharmacological inhibition or gene knockout drives a reduction in the efflux rate, compromising the regulation of internal pH (Lopez-Charcas et al., 2022). Observations suggest that the colocalization of Nav1.5 and NHE1 in colorectal cancer and breast cancer cells supports cancer invasiveness (Brisson et al., 2013; Amith and Fliegel, 2017; Lopez-Charcas et al., 2022). ii) Na^+^/HCO3^−^ cotransporter (NBC). NBC transporters are able to load or extrude protons from the cell via sodium-bicarbonate transport. NBCe1, a type of NCB transporter, is present in pancreatic cancer cells and functions as an acid extruder that supports tumoral growth and metastasis (Cappellesso et al., 2022). iii) Monocarboxylate transporters (MCT). MCT are monocarboxylate transporters that extrude protons together with a short fatty acid, such as lactic acid or pyruvate (Pértega-Gomes et al., 2011). MCT4 is overexpressed in prostate cancer and is linked with a poor prognosis (Pértega-Gomes et al., 2011). iv) Cl^−^/HCO_3_^−^ anion-exchanger (AE). AEs are chloride-bicarbonate transporters, where AE2a overexpression is associated with an incremented cell malignancy in colorectal cancer cells (Khosrowabadi et al., 2021), and v) Vacuolar (H^+^)-ATPases. V-ATPase is a proton pump that promotes the transport of protons in disfavor of their chemical gradient, coupled to ATP consumption. V-ATPases are expressed in the plasmatic surface of breast cancer cells, where they support a metastatic phenotype (Sennoune et al., 2004). These molecular entities contribute to the acidification of the tumor microenvironment, as well as the intracellular alkalinization of cancer cells, triggering many pH-dependent processes that promote the proliferation and malignancy of some types of solid tumors, which were well reviewed elsewhere (Boedtkjer and Pedersen, 2020). Although the role of these proton transporters is well studied, research in recent decades has placed the voltage-sensitive proton channel Hv1 as a fundamental pH regulator in some types of cancers, acquiring almost as much prominence as the previously mentioned intracellular pH regulators. Here we summarize evidence related to the expression of Hv1 channel in a diversity of tumoral cells, with special focus on solid tumors, which are consistent with the idea that this protein channel contributes to maintaining the cancer physiopathology, including it as a predictor of incidence and cancer malignity. In the following section, the features of the tumoral phenotype are depicted and discussed together with the role of Hv1 in different tumor types:

### 5.1 The tumor microenvironment is where the tumorigenic process mainly occurs

**FIGURE 3:**
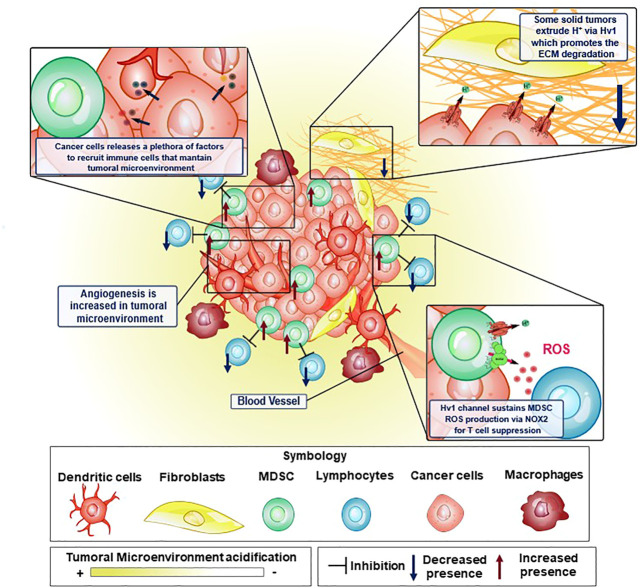
Elements of the tumor microenvironment and how Hv1 channel is crucial to sustain some malignant features in cancer. Tumoral microenvironment is constituted by several cellular elements, where immune system cells are key to maintain the tumoral microenvironment conditions to promote cancer cell development. In some of these cellular elements, the Hv1 channel has been shown to possess an important role. Proton extrusion through Hv1 promotes local acidification of the TME, which in turn, has been shown to induce the activation of extracellular matrix degrading proteases favoring cancer cell migration. On the other hand, MDSC are strongly immunosuppressive, preventing and interfering with tumor elimination by T-cells. This immunosuppression is led by ROS production via NOX2 enzymatic complex, a process that is sustained thanks to the functional coupling between NOX2 and Hv1 channel. Moreover, cancer cells release tumoral factors that maintain the features of the tumoral microenvironment. Examples of these are VEGF, HIF, GM-CSF, TNF-α, IL-12, IL-10, TGF-β. GM-CSF recruits MDSC to the tumoral microenvironment and maintains its strongly immunosuppressive phenotype.

Hv1 seems to be significant in the TME of solid tumors. The TME is composed of non-tumoral, tumoral and multiple immune cell types, accompanied by an exuberant surrounding vascularization (Figure 3). These cells live in an extracellular space with both low partial pressure of oxygen and low circumventing metabolites (Jin and Jin, 2020). Adapting to this nature, the TME exhibits an acidic pH and is a strongly immunosuppressive phenotype, inhibiting the anti-tumoral function of the host. All these important aspects of the TME are detailed below:

#### 5.1.1 EMC remodeling processes promotes the TME

It is widely reported that ECM remodeling is a crucial process in cancer development (Winkler et al., 2020). These events associated with ECM modification allow the successful establishment of the tumor microenvironment, enhancing tumor malignancy. The processes associated with EMC remodeling during cancer development range from physical to biochemical changes (Egeblad et al., 2010; Kai et al., 2019; Winkler et al., 2020), and are mainly classified as: 1) ECM deposition, which corresponds to a set of changes in the composition and abundance of ECM components; 2) post-translational level modifications; and **3)** degradation by proteolytic enzymes (Kai et al., 2019; Winkler et al., 2020). All these modifications together dramatically affect the physical integrity as well as the biochemical properties of the ECM, thus causing the elimination of certain structural and anchoring constraints, promoting cell migration, and the formation of neovascularization in the TME, mechanisms that were well reviewed (Egeblad et al., 2010; Kai et al., 2019; Winkler et al., 2020). In the TME, there are enzymes that degrade and cleave the proteins that compose the ECM. These enzymes, such as matrix metalloproteinases (MMPs), can be expressed by tumoral cells in the tumor itself (Bergers et al., 2000; Yang et al., 2004; Vihinen et al., 2005; Kessenbrock et al., 2010; Gialeli et al., 2011; Mehner et al., 2014; Yousef et al., 2014), It has been observed that MMP9, is one of the most important proteolytic enzymes in ECM remodeling in various types of cancers (Bergers et al., 2000; Huang et al., 2002; Yang et al., 2004; Ahn and Brown, 2008; Yousef et al., 2014; Joseph et al., 2020). Interestingly, some reports show that the participation of MMP9 is positively modulated by extracellular acidification at several points, promoting their release and activity (Glunde et al., 2003; Wang et al., 2012; Christensen and Shastri, 2015; Ordway et al., 2021). MMP9 is secreted in an inactive form as pro-enzyme, and is activated by cleavage of the pro-domain (Van Wart and Birkedal-Hansen, 1990). The release of some of its activators, such as MMP2 and certain cathepsins, are favored by extracellular acidification (Glunde et al., 2003; Wang et al., 2012; Christensen and Shastri, 2015; Ordway et al., 2021).

#### 5.1.2 Aberrant vascularization and sustained hypoxia are present at the TME

Diverse mechanisms are proposed to explain the vessel phenotype around the TME (Figure 3), such as the expansion of the vascular network by formation of capillary bridges and/or endothelial sprouts, the insertion into the lumen of pre-existing vessels in interstitial tissue and by endothelial cellular precursors migrating from peripheral blood or bone marrow into the TME, all leading to the lining of tumorigenic vessels into the endothelial tissue (Carmeliet and Jain, 2000). The previous processes are tightly regulated by several cellular signaling systems. The distribution and ramification of these capillaries are abnormal, aberrant and poorly present deeper into the inner core of solid tumors (Goel et al., 2011). This phenotype does not favor the nutrient and oxygen supply to cells. However, tumoral cells do survive in such an adverse environment by exploiting the upregulation of hypoxia-inducible factor (HIF) and its signaling, leading to a cellular phenotype which can survive in low-oxygen conditions, triggering dormancy, enhanced heterogeneity of tumors, cell stemness and metabolic reprogramming (Qiu et al., 2017). The HIF, normally degraded in normoxia, is stabilized in hypoxia, being able to act as a transcriptional factor, inducing the expression of genes involved in the metabolic adaptation of the tumoral cells to hypoxic conditions, including the expression of the Vascular endothelial growth factor (VEGF) (RamaKrishnan and Sankaranarayanan, 2016), which leads to an aberrant vascularization network, and switching the main energetic obtention outcome from oxidative phosphorylation to glycolysis. The activation of this metabolic re-programming involves the induction of the expression of glucose transporters, hexokinase 2 and some isoforms of PFK2, at the same time reducing cellular respiration by regulating the expression of cytochrome c oxidase and inhibiting the mitochondrial biogenesis (Denko, 2008). Despite tumor cells having a high glycolytic rate that causes an increased intracellular proton production, the function of several proton extruders (such as NHE1, NBCs, or Hv1, to mention) that expel these protons, maintain their internal pH to generally stay within the range of normal physiological values (between 7.2 and 7.4, according to Webb et al., 2011. This, in turn, can lead to acidification of the extracellular medium, in some cases such as sarcomas or adenocarcinomas, even going to pH 6.7 or lower (Thistlethwaite et al., 1985). In order to maintain the intracellular pH homeostasis in solid tumors, and avoid malfunctioning of the molecular machinery caused by harmful levels of intracellular acidification, the tumoral cells use transmembrane molecules that can extrude the excess protons from the cytosol, alkalinizing the intracellular pH and contributing to the acidification of the extracellular side (Le Floch et al., 2012; Wang et al., 2012; 2013b; Asuaje et al., 2017; Boedtkjer and Pedersen, 2020; Ventura et al., 2020) (Figure 3).

#### 5.1.3 Host immune anti-tumoral system is inhibited by immunosuppressive TME

**FIGURE 4:**
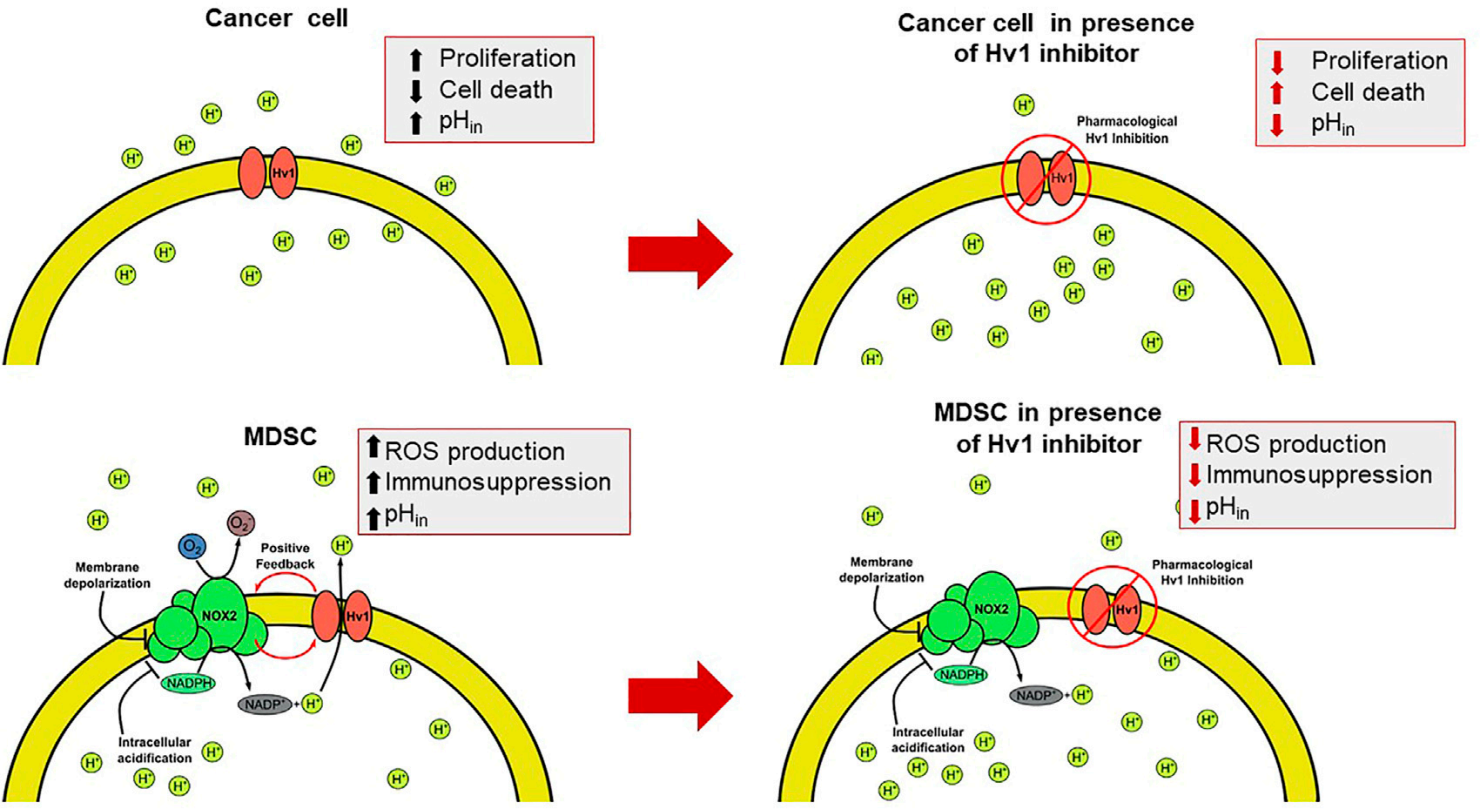
The intervention of the Hv1 function as a feasible therapeutic strategy for circumventing the TME. The presence of the Hv1 channel in cancer cells could be helpful to control the cytosolic pH. Once blocked, intracellular acidification could be harmful for tumors. On the other hand, in MDSC, Hv1 allows the sustained production of ROS, which give rise to the immunosuppressive function of MDSC. Then, the inhibition of Hv1 could diminish its immunosuppressive function. Both effects together could induce a decrease in tumor size.

The TME exhibits both an impaired recognition by the host immune system and a strong immunosuppressive activity associated principally with aberrant myelopoiesis. Some tumoral factors, like Granulocyte-macrophage colony-stimulating factor (GM-CSF), Interleukin 4 (IL-4), or VEGF, to mention some, are secreted from tumoral cells and can signal hematopoietic precursors to change their differentiation pathway (Figure 3) (Umansky et al., 2016), effectively reducing their differentiation to dendritic cells, leading to multiple undifferentiated populations, most notably, cells with an immunosuppressive phenotype, such as myeloid-derived suppressor cells (MDSC). MDSC strongly inhibits the activity of T lymphocytes (Figure 3). In cancer contexts, they are recruited into the TME where these cells exert several mechanisms to inhibit the T-cell response which can operate either individually or collectively (Serafini, 2013). These immunosuppressive strategies use four different mechanisms: i) the depletion of amino acids essentials for the activation of T lymphocytes, such as arginine and cysteine; ii) the damage of the viability and trafficking of T cells; iii) the recruitment and proliferation of regulatory T-cells and iv) the production of ROS and RNS via NOX2 (Kusmartsev and Gabrilovich, 2006; Corzo et al., 2009; Gabrilovich et al., 2012). It has been reported that the main mechanism of immunosuppression associated with tumor microenvironment by the MDSC corresponds to ROS and RNS production led by the NOX2 complex (Lu and Gabrilovich, 2012). The mechanism behind the immunosuppression mediated by these free radicals consists of the direct modification of the T-cell receptor (Nagaraj et al., 2007). Recently, it was reported that the Hv1 channel plays a key role in sustaining the ROS-mediated immunosuppression produced by MDSC (Alvear-Arias et al., 2022) (Figure 4). Here, the Hv1 channel allows the sustained production of ROS by the MDSC via functional coupling with NOX2, similarly as reported in proinflammatory immune cells. Interestingly, the prolonged exposure of MDSCs to Hv1 inhibitors seems to impair its immunosuppressive phenotype (Figure 4) (Alvear-Arias et al., 2022). Since the MDSC is constantly producing ROS, Hv1 is always extruding protons to the extracellular side of the membrane, effectively contributing to the acidification of the TME. Interestingly, it has been observed that MDSCs are rich in metalloprotease (MMP) 9 (Yang et al., 2004). MMPs are a family of matrix-degrading enzymes that are involved in extracellular matrix (ECM) remodeling, thus promoting malignancy-associated processes such as angiogenesis and tumor cell migration (Bergers et al., 2000; Ahn and Brown, 2008; Kessenbrock et al., 2010; Mehner et al., 2014). The release of MMPs is promoted by the extracellular acidification (Wang et al., 2011; 2012), that characterizes the tumor microenvironment, a process in which Hv1 may also be involved.

### 5.2 Hv1 is a pro-tumoral molecule in tumor biology

The role of the voltage-gated proton channel in tumor biology has begun to emerge during the past decade. The acid-base state and pH control is not only important for healthy cells, since multiple studies have revealed that pH regulation in cancer cells and the tumor microenvironment is deregulated, which greatly favors the development of the disease. Indeed, one of the striking features of cancer is the inversion of intracellular and extracellular pH values compared to normal physiological parameters. The increase in intracellular proton concentration ([H+]in, pH_in_) of a cancer cell is achieved while exhibiting classic high glycolytic rate, which is increased to satisfy its high energy demand. This high glycolytic metabolism is characterized by a high consumption of glucose that produces an excessive output of lactate and H+ (Helmlinger et al., 2002). All these proton equivalents produced in the cytosol are detrimental to tumoral cells, as the acidic pH can inhibit their glycolytic metabolism and compromise their survival and proliferation (Boedtkjer and Pedersen, 2020). Beneficially to the tumoral cells, all the overproduced proton equivalents are extruded by a diverse and selective group of molecules that allow the protons to be transported out of the cell, such as the Na+, HCO3 co-transporter NBCn1, the Na+/H + -exchanger, H + -ATPases, to mention (Boedtkjer and Pedersen, 2020). These can enable an increase in the intracellular pH, but less or very little is known about channel intervention regarding proton transport across membranes of tumoral cells. Currently, there has been an increasing number of studies which reports that the Hv1 proton channel has a key role in proton extrusion in tumor cells, relating their overexpression in some cases to tumor malignancy (Wang et al., 2011; 2012; 2013b; Yu et al., 2014; Asuaje et al., 2017) (Figure 4). There are some systemic tumors where Hv1 is expressed, such as:

#### 5.2.1 Leukemia

In Jurkat T cells the Hv1 expression is tightly related to intracellular pH homeostasis. The perturbation of its function by the classic inhibitor Zn^2+^, or the more selective ClGBI, besides leading to a significant reduction in the current intensity of these cells when measured by whole-cell patch clamp, it also conducted to a reduction in the intracellular pH_i_ both in the short and long term (Asuaje et al., 2017), strongly suggesting that the proper function of Hv1 at Jurkat T cells is important and relevant to intracellular pH_i_ homeostasis in a concentration-dependent and time-dependent manner. Crucial to the cell’s fate, manipulation of Hv1 was observed to be strictly related to it: the inhibition of Hv1 with 200 or 800 µM ClGBI increased the proportion of cells positive for Annexin V and for propidium iodide proportionally to the time of incubation with the drug, suggesting that the selective blockade of Hv1 in Jurkat T cells could lead to the induction of cell death through the apoptosis process (Asuaje et al., 2017). Interestingly, the histamine receptor H1 antagonist diphenhydramine (DPH) showed a marked reduction in the intensity of proton currents in patch clamp in whole-cell configuration of Jurkat T cells (Asuaje et al., 2018), while histamine application showed no reduction of the currents. Hence, the presence of DPH induces an impairment of viability and overall intracellular acidification. Together with the previous, an induction of viability impairment and acidification of the intracellular pH was observed.

#### 5.2.2 Lymphoma

In chronic lymphocytic leukemia (CLL) and malignant B cells, an overexpression of the short isoform known as HCVN1s was found (Hondares et al., 2014). Specifically, this isoform is 20 amino acids shorter than the conventional Hv1 protein and varies particularly in the length of its N-terminal (Capasso et al., 2010). It was observed that this isoform responds more strongly to phosphorylation via PKC with respect to canonical Hv1 or HVCN1L (as the authors refer to it), which induces the phenomenon known as “enhanced gating mode” (Bánfi et al., 1999; Decoursey, 2003). The phosphorylation site corresponds to Thr9 in the short isoform and Thr29 for the canonical channel (Musset et al., 2010; Hondares et al., 2014), and possibly the structural differences that emerge from the shortening of the N-terminal ensure a more sensitive gating to phosphorylation in the short isoform. Thus, Hondares et al., reported that overexpression of this short isoform improves B cell receptor signaling, increases proliferation, and promotes chemokine-dependent migration, which confers greater advantages to the malignant B cells (Hondares et al., 2014).

Although there is evidence regarding systemic tumors and Hv1, most of the literature connecting Hv1 to cancer is linked to Hv1 in solid tumors. In the following section.

#### 5.2.3 Glioma

Congruent results were found in glioma cell lines, demonstrating that one of the important findings regarding Hv1 is that the protein seems to be related to the capacity of the tumor for metastasis. When examining the highly metastatic glioma SHG-44, a greater expression of Hv1 was found than when compared to a glioma with lower capacity for metastasis (U-251) (Wang et al., 2013b). It was then proposed that Hv1 presents a role on invasion and migration by regulating intracellular pH and gelatinase activity, to the point in which inhibition of the channel promotes apoptosis on SHG-44 cells.

#### 5.2.4 Glioblastoma

In human glioblastoma multiforme (GBM), a particularly deadly cancer, a functional expression of Hv1 was also detected, and when inhibited, it has been shown to increase extracellular pH, lowering tumor proliferation rate and promoting astrocyte activity and response (Ribeiro-Silva et al., 2016). It is then clear that detection of Hv1 expression could allow for a diagnosis of malignancy of several tumors, where higher expressions of the channel are linked to migration and invasion from the tumor.

#### 5.2.5 Colorectal cancer

In colorectal cancer, Hv1 is overexpressed, and has been proposed as a biomarker for colorectal cancer development (Wang et al., 2013a; Yu et al., 2014). Besides, this increase of the Hv1 expression has been associated with sedentary behavior, especially in the long-term. The channel has been associated with a clear poor prognosis related to a higher expression of Hv1 in colorectal patients, exhibiting both shorter overall survival and recurrence-free survival. Furthermore, these patients exhibited an early recurrence when compared to patients with lower Hv1 levels (Wang et al., 2013a). The silencing and inhibition of the proton channel *in vitro,* in colorectal cancer cell line SW620, mitigated its migration and invasion and suppressed proton extrusion (Wang et al., 2013a).

#### 5.2.6 Breast cancer

The first studies that positioned Hv1 as an important proton extruder involved in tumor malignancy were those published by Wang et al., who reported that Hv1 is expressed in breast cancer cell lines (Wang et al., 2012). At this time, it was observed that the differences in the expression levels of the Hv1 channel are correlated to the invasion and migration (Hanahan and Weinberg, 2000; 2011) (both hallmarks of tumor malignancy) of the two breast cancer cell lines: MDA-MB-231, characterized by being highly invasive with high migratory capacity, and the MCF-7 line, which is poorly metastatic, i.e., possesses a low migratory capacity and low invasive capacity (Wang et al., 2011). Subsequently, they evaluated how the decrease in Hv1 expression affected these characteristics using siRNA-Hv1. Interestingly, the expression of the Hv1 protein is increased in MDA-MB-231 in comparison with MCF-7. In addition, Hv1 suppression with si-RNA drastically decreased invasion and migration in MDA-MB-231, suggesting that the expression of this proton channel is related to a pattern of malignancy in human breast cancer cells (Wang et al., 2012). The authors studied the cell migration by scratch assays of MDA-MB-231 cells and siRNA Hv1 MDA-MB-231 cells, observing that the knock-down of Hv1 slowed down the migration of the cells. In a further work published by Bare et al., by means of an *in-vivo* modeling of tumor growth showed that the knock-out (KO) of Hv1 in MDA-MB-231 cells contributed to a reduction in its tumor size when injected in mice, compared to its wild-type (WT) counterpart (Bare et al., 2020). It has been shown in the tumorigenic MCF-7 breast cancer cell line, that the time-dependent inhibition of Hv1 with 10 µM ClGBI led to an acidification of its intracellular pH, suggesting an interesting role in intracellular pH regulation in MCF-7 cells. This observation was also made in MDA-MB-231 cells but not in non-tumorigenic MCF-10A breast cell lines (Ventura et al., 2020). The viability of MCF-10A, MCF-7 and MDA-MB-231 cells were studied by pharmacological inhibition with ClGBI, showing no differences in MCF-10A but a dramatic decrease in MCF-7 and MDA-MB-231 cell viability. Recovery experiments were also performed, with no changes observed for MCF-10A cells, a slight recuperation of cell viability in MCF-7 cells but a compromised cell viability in MDA-MB-231 cells, suggesting that Hv1 in these tumorigenic breast-cancer lines does influence their cell viability related to the cytosolic proton extrusion (Ventura et al., 2020). 5 and 10 µM ClGBI were applied for 10 h on MCF-10A, MCF-7 and MDA-MB-231 cultures to study the effect of Hv1 inhibition on the cell-cycle distribution. No effect was detected either in MCF-10A or MCF-7 cells, but a clear diminishing in the percent of the cell cycle distribution at G0/G1 was observed in MDA-MB231 cells loaded with 10 µM ClGBI. No statistical differences were observed at the S phase and a clear increase of the percent of cell cycle distribution was evidenced in MCF-7 cells and MDA-MB-231 cells with 10 µM ClGBI on G2/M interphase, while no effect was noted in MCF-10A. Metaphase-arrested cells were also evaluated, showing a statistically significant rise in the percentage of tumorigenic mitosis-arrested breast cells and no effect on the non-tumorigenic cell line when 10 µM ClGBI was added (Ventura et al., 2020). The clonogenic capability of the previous breast cell line was put to the test over a wide range up to 20 µM ClGBI. In all cell lines, the higher the concentration of ClGBI, the lower the clonogenic capability. Its recovery was tested, showing a clonogenic recovery close to 30% for 10 µM ClGBI and below 10% for 15 and 20 µM ClGBI in non-tumorigenic MCF-10A cells. Similarly, MCF-7 and MDA-MB-231 results were below 10% for 10 µM ClGBI and near zero for both 15 and 20 µM ClGBI, suggesting great clonogenic modulation of tumorigenic breast cancer cell lines MCF-7 and MDA-MB-231 by ClGBI (Ventura et al., 2020). A reduced viability was evidenced in a three-dimensional cell culture of MCF-7 cells when diverse µM ClGBI concentrations were imposed, reaching nearly 80% of reduction at 1 mM ClGBI. Interestingly, the authors reported a differential expression of Hv1 composed of both the long canonic Hv1 and a short Hv1 isoform over the three breast cell lines studied so far. While the MCF-10A cells appear to express exclusively the long isoform, for the MCF-7 cell line a mixture of nearly 50% of the total for each isoform (long and short) is suggested and a way more polarized expression of the short isoform of Hv1 in comparison to the long isoform of Hv1 in MDA-MB-231, suggesting that these tumorigenic cells rely on the expression of diverse isoforms of Hv1 regarding its tumorigenic capability, where apparently a higher proportion of the short isoform of Hv1 is expressed in the most metastatic cell type compared to its longer isoform. How does the presence of the Hv1 channel regulate the malignancy of breast cancer? The secretion and activation of some proteases that degrade collagens of the ECM, is highly regulated by pH. Wang et al., reported in previous works that the activity of certain proteases, like the matrix 2 metalloproteinase 2 (MMP-2) and the matrix metallopeptidase 9 (MMP-9) are related to the degradation of the ECM promoting tumor cell migration, invasion and metastasis, a phenomenon that has also been reported in other types of cancers (Bergers et al., 2000; Ahn and Brown, 2008; Gialeli et al., 2011; Wang et al., 2012; Mehner et al., 2014; Joseph et al., 2020, please be referred to Table 2 for more examples including their references). Extracellular acidification favors the extracellular cell matrix degradation by altering the nature of the proteins that compose the matrix, but also by promoting the secretion and activation of proteases. ECM modification facilitates the migration of cancer cells and angiogenesis (Figure 3) (Egeblad et al., 2010; Kessenbrock et al., 2010; Winkler et al., 2020). It has been observed that the Hv1 silencing by siRNA reduces the MMP-2 expression in MDA-MB-231 highly metastatic cells (Wang et al., 2012), which suggests that the activity of Hv1 is supporting invasion and metastasis in breast cancer cells, by maintaining the perfect levels of both intracellular and extracellular pH for secretion, activation and distribution of proteases involved in extracellular matrix digestion such as MMP-2. Recent studies have linked the elevated MMP9 enzyme levels with poor prognosis in breast cancer patients (Joseph et al., 2020), thus establishing an interesting correlation between the Hv1 channel upregulation, high levels of MMP9 expression in the breast cancer TME and poor survival in breast cancer patients.

**TABLE 2:** Presence of the Hv1 channel in tumoral cells.

Type of cancer	Effect	References
Breast Cancer	Hv1 as a biomarker of malignancy and its inhibition deters tumor cell viability	Wang et al. (2011), Wang et al. (2012), Bare et al. (2020), Ventura et al. (2020)
Colorectal Cancer	The expression of Hv1 is increased and its prevalence is linked to poor prognosis	Wang et al. (2013a), Yu et al. (2014)
Leukemia	The inhibition of Hv1 induces death by apoptosis on a leukemia model	Asuaje et al. (2017), Kawai et al. (2018)
Glioma	The inhibition of Hv1 produces death by apoptosis on highly metastatic glioma cells and reduces tumor volume *in vivo*	Wang et al. (2013b)
Glioblastoma	Inhibition of Hv1 reduces cell viability and migration on glioblastoma multiforme	Ribeiro-Silva et al. (2016)
Lymphoma	Malignant B-cells express a short isoform of Hv1 that confers a proliferative and migratory advantage	Hondares et al. (2014)

Nevertheless, it is certain that Hv1 seems to be a pro-tumoral molecule, as it is exploited by tumoral cells of diverse tissues in order to regulate their intracellular pH that is in constant acidification mainly because of the metabolic reprogramming induced by the Warburg effect. All the previous tumoral cell lines where Hv1 is present are of public health interest, including aggressive cancer cell types such as glioblastoma, lymphoma and breast cancer cells. Thus, Hv1 could serve as a marker of tumor progression or aggressiveness of certain tumoral cell lines, and secondly, the intervention of Hv1 in tumoral biology, such as inhibition or genetic deletion, may lead to a direct perturbation of cancer cells.

## 6 Hv1 channel as a novel and promising therapeutic target in tumoral progress

Although there are several promising therapeutic approaches to fight the development of tumor tissue growth, such as immunotherapy, to date, there is still no transversal anti-tumoral strategy effective against tumoral progression (Dunn et al., 2002; Stagg et al., 2007; Yang, 2015; Esfahani et al., 2020). Notwithstanding the above, as we reviewed previously, a common feature among cancer cells is their enhanced cytosolic proton production and its extrusion by multiple molecules that enable the perfect cytosolic pH conditions for them to thrive on. Much evidence has increased the scientific interest in the role of the Hv1 proton channel as an anti-tumoral therapeutic target, as they are present in a broad set of cancer types, contributing to the progression of the neoplastic process by enhancing the survival and the metabolism of tumoral cells, alkalinizing its cytosol (See section above). It may appear Hv1 could be an important pro-tumoral molecule of solid tumors. One of the most common types of solid tumors are represented by carcinomas (https://www.cancer.gov/about-cancer/understanding/what-is-cancer, last visited in December 2022), such as epithelial cells colonized by skin tumoral cells, or skin cancer. As solid tumors are a central part of tumoral biology, Hv1 may be a very promising pro-tumoral candidate for anti-tumoral therapy. Moreover, Hv1 has not only been observed in tumor cells, but it is also expressed in immunosuppressive cells promoted by the TME such as MDSC (Alvear-Arias et al., 2022). As mentioned, these cells exert their immunosuppression through Hv1-mediated ROS production in the tumor microenvironment, preventing immune-mediated destruction and promoting tumor survival, colonization, proliferation, among others. This evidence contributes to the understanding of the role of Hv1 in the TME further than its exclusive role in tumoral cells, now including its participation in MDSC and immunosuppression.

Hv1 knockout in mice leads to an incremented number of activated T cells (CD4^+^ and CD8^+^) in unchallenged and challenged conditions (infection with LCMV), in comparison with WT mice (Sasaki et al., 2013). The authors conclude that although there is a link between a high number of activated T cells and auto-immunity disease, there is still a need for further evidence in order to confirm if the lack of Hv1 channels is the main inductor of autoimmunity. Recently, the presence of Hv1 channels in T lymphocytes, and that loss of Hv1 in T lymphocytes leads to a metabolic reprogramming and an impaired activation of T lymphocytes that could affect their priming stage, possibly affecting their anti-tumor response (Coe et al., 2022). They evidenced that 200 μM of ClGBI diminished CD8^+^ T cells pH_i_, viability and incremented apoptosis and necrosis, but haven’t investigate their anti-tumoral function in these cell types up to date. The previous experiment does suggest that the addition of ClGBI could alter the number of viable T lymphocytes. Although there is still a need for anti-tumoral response studies regarding inhibition or deletion of Hv1 in these cells, it appears that the inhibition of Hv1 in the tumoral system with ClGBI could affect the anti-tumoral response of T lymphocytes (Coe et al., 2022). On the other hand, there is no evidence regarding inhibition of Hv1 channels in T lymphocytes with zinc ion. It could be very interesting to experimentally perform inhibition of T lymphocytes Hv1 with zinc ion, as in some reports, it is mentioned that zinc ion addition to T cell cultures enhanced their proliferation and their signaling. Somehow, this could alleviate the decrease in viability observed when inhibiting Hv1 T lymphocytes with ClGBI (Yu et al., 2011; Rosenkranz et al., 2016).

We encourage investigators to inhibit Hv1 with zinc ion, especially *in vivo*, as it is simpler, and promotes T lymphocyte signaling and proliferation, it could be further efficient in tumor treatment, in comparison with specific Hv1 channel inhibitors (ClGBI).

All these findings gathered in this work points to Hv1 as an important promoter of tumoral progress. The number of studies suggesting that this channel could be a pH regulator with pro-tumorigenic activity has expanded over the years, expressed in several cellular elements that make up the tumor microenvironment, positioning it as a marker of tumor malignancy. Thus, this protein could be a common characteristic in many types of solid tumors, which could generate a supplementary therapy to the existing ones. To date, there are few specific inhibitors of the Hv1 proton channel, and much progress is required in the design and synthesis of drugs aimed at inhibiting the activity of this channel, however, this could have interesting results directed towards the strategy of growth control and development, focused on attacking the fine pH balance that characterize cancer cells.

Future pre-clinic and clinical positive results regarding Hv1 blocking without suppression of immune system could be a promising complementary anti-tumoral therapy.

## References

[B1] Ahn G. O. Brown J. M. (2008). Matrix metalloproteinase-9 is required for tumor vasculogenesis but not for angiogenesis: Role of bone marrow-derived myelomonocytic cells. Cancer Cell 13, 193–205. 10.1016/j.ccr.2007.11.032 18328424 PMC2967441

[B2] Alabi A. A. Bahamonde M. I. Jung H. J. Kim J. Il Swartz K. J. (2007). Portability of paddle motif function and pharmacology in voltage sensors. Nature 450, 370–375. 10.1038/nature06266 18004375 PMC2709416

[B3] Alvear-Arias J. J. Carrillo C. Villar J. P. Garcia-Betancourt R. Peña-Pichicoi A. Fernandez A. (2022). Expression of H v 1 proton channels in myeloid-derived suppressor cells (MDSC) and its potential role in T cell regulation. Proc. Natl. Acad. Sci. 119, e2104453119. 10.1073/pnas.2104453119 35377790 PMC9169626

[B4] Amith S. R. Fliegel L. (2017). Na+/H+ exchanger-mediated hydrogen ion extrusion as a carcinogenic signal in triple-negative breast cancer etiopathogenesis and prospects for its inhibition in therapeutics. Semin. Cancer Biol. 43, 35–41. 10.1016/j.semcancer.2017.01.004 28104391

[B5] Armstrong C. M. Hollingworth S. (2018). A perspective on Na and K channel inactivation. J. Gen. Physiol. 150, 7–18. 10.1085/jgp.201711835 29233885 PMC5749110

[B6] Asuaje A. Martín P. Enrique N. Zegarra L. A. D. Smaldini P. Docena G. (2018). Diphenhydramine inhibits voltage-gated proton channels (Hv1) and induces acidification in leukemic Jurkat T cells- New insights into the pro-apoptotic effects of antihistaminic drugs. Channels (Austin) 12, 58–64. 10.1080/19336950.2017.1331799 28514187 PMC5972794

[B7] Asuaje A. Smaldini P. Martín P. Enrique N. Orlowski A. Aiello E. A. (2017). The inhibition of voltage-gated H+ channel (HVCN1) induces acidification of leukemic Jurkat T cells promoting cell death by apoptosis. Pflugers Arch. Eur. J. Physiol. 469, 251–261. 10.1007/s00424-016-1928-0 28013412

[B8] Babior B. M. Kipnes R. S. Cumvu J. T. (1973). Biological defense mechanisms. The production by leukocytes of superoxide, a potential bactericidal agent. CONCISE Biol. Def. Mech. 52, 741–744. 10.1172/JCI107236 PMC302313 4346473

[B9] Bánfi B. Schrenzel J. Nüsse O. Lew D. P. Ligeti E. Krause K. H. (1999). A novel H+ conductance in eosinophils: Unique characteristics and absence in chronic granulomatous disease. J. Exp. Med. 190, 183–194. 10.1084/jem.190.2.183 10432282 PMC2195580

[B10] Bare D. J. Cherny V. V. DeCoursey T. E. Abukhdeir A. M. Morgan D. (2020). Expression and function of voltage gated proton channels (Hv1) in MDA-MB-231 cells. PLoS One 15, e0227522–e0227527. 10.1371/journal.pone.0227522 32374759 PMC7202653

[B11] Bayrhuber M. Maslennikov I. Kwiatkowski W. Sobol A. Wierschem C. Eichmann C. (2019). Nuclear magnetic resonance solution structure and functional behavior of the human proton channel. Biochemistry 58, 4017–4027. 10.1021/acs.biochem.9b00471 31365236

[B12] Berger T. K. Fußhöller D. M. Goodwin N. Bönigk W. Müller A. Dokani Khesroshahi N. (2017). Post-translational cleavage of Hv1 in human sperm tunes pH- and voltage-dependent gating. J. Physiol. 595, 1533–1546. 10.1113/JP273189 27859356 PMC5330862

[B13] Berger T. K. Isacoff E. Y. (2011). The pore of the voltage-gated proton channel. Neuron 72, 991–1000. 10.1016/j.neuron.2011.11.014 22196334 PMC3244940

[B14] Bergers G. Brekken R. McMahon G. Vu T. H. Itoh T. Tamaki K. (2000). Matrix metalloproteinase-9 triggers the angiogenic switch during carcinogenesis. Nat. Cell Biol. 2, 737–744. 10.1038/35036374 11025665 PMC2852586

[B15] Bezanilla F. (2018). Gating currents. J. Gen. Physiol. 150, 911–932. 10.1085/jgp.201812090 29941430 PMC6028497

[B16] Boedtkjer E. Pedersen S. F. (2020). The acidic tumor microenvironment as a driver of cancer. Annu. Rev. Physiol. 82, 103–126. 10.1146/annurev-physiol-021119-034627 31730395

[B17] Bradley P. Misura K. M. S. Baker D. (2005). Biochemistry: Toward high-resolution de novo structure prediction for small proteins. Science 309, 1868–1871. 10.1126/science.1113801 16166519

[B18] Brisson L. Driffort V. Benoist L. Poet M. Counillon L. Antelmi E. (2013). NaV1.5 Na⁺ channels allosterically regulate the NHE-1 exchanger and promote the activity of breast cancer cell invadopodia. J. Cell Sci. 126, 4835–4842. 10.1242/jcs.123901 23902689

[B19] Capasso M. Bhamrah M. K. Henley T. Boyd R. S. Langlais C. Cain K. (2010). HVCN1 modulates BCR signal strength via regulation of BCR-dependent generation of reactive oxygen species. Nat. Immunol. 11, 265–272. 10.1038/ni.1843 20139987 PMC3030552

[B20] Cappellesso F. Orban M. P. Shirgaonkar N. Berardi E. Serneels J. Neveu M. A. (2022). Targeting the bicarbonate transporter SLC4A4 overcomes immunosuppression and immunotherapy resistance in pancreatic cancer. Nat. Cancer 3, 1464–1483. 10.1038/s43018-022-00470-2 36522548 PMC9767871

[B21] Carmeliet P. Jain R. K. (2000). Angiogenesis in cancer and other diseases. Nature 407, 249–257. 10.1038/35025220 11001068

[B22] Carmona E. M. Fernandez M. Alvear-Arias J. J. Neely A. Larsson H. P. Alvarez O. (2021). The voltage sensor is responsible for ΔpH dependence in Hv1 channels. Proc. Natl. Acad. Sci. U. S. A. 118, 20255561188–e2025556127. 10.1073/pnas.2025556118 PMC8126849 33941706

[B23] Carmona E. M. Larsson H. P. Neely A. Alvarez O. Latorre R. Gonzalez C. (2018). Gating charge displacement in a monomeric voltage-gated proton (H v 1) channel. Proc. Natl. Acad. Sci. 115, 9240–9245. 10.1073/pnas.1809705115 30127012 PMC6140481

[B24] Carr D. W. Acotf T. S. (1989). Intracellular pH regulates bovine sperm motility and protein phosphorylation. Biol. Reprod. 920, 907–920. 10.1095/biolreprod41.5.907 2624855

[B25] Carr D. W. Acott T. S. (1984). Inhibition of bovine spermatozoa by caudal epididymal fluid: I. Studies of a sperm motility quiescence factor. Biol. Reprod. 30, 913–925. 10.1095/biolreprod30.4.913 6329336

[B26] Cassetta L. Baekkevold E. S. Brandau S. Bujko A. Cassatella M. A. Dorhoi A. (2019). Deciphering myeloid-derived suppressor cells: Isolation and markers in humans, mice and non-human primates. Cancer Immunol. Immunother. 68, 687–697. 10.1007/s00262-019-02302-2 30684003 PMC6447515

[B27] Cherny V. V. Markin V. S. DeCoursey T. E. (1995). The voltage-activated hydrogen ion conductance in rat alveolar epithelial cells is determined by the pH gradient. J. Gen. Physiol. 105, 861–896. 10.1085/jgp.105.6.861 7561747 PMC2216954

[B28] Cherny V. V. DeCoursey T. E. (1999). pH-Dependent inhibition of voltage-gated H+ currents in rat alveolar epithelial cells by Zn2+ and other divalent cations. J. Gen. Physiol. 114, 819–838. 10.1085/jgp.114.6.819 10578017 PMC2230650

[B29] Cherny V. V. Murphy R. Sokolov V. Levis R. A. DeCoursey T. E. (2003). Properties of single voltage-gated proton channels in human eosinophils estimated by noise analysis and by direct measurement. J. Gen. Physiol. 121, 615–628. 10.1085/jgp.200308813 12771195 PMC2217352

[B30] Christensen J. Shastri V. P. (2015). Matrix-metalloproteinase-9 is cleaved and activated by Cathepsin K. BMC Res. Notes 8, 322–328. 10.1186/s13104-015-1284-8 26219353 PMC4518881

[B31] Coe D. Poobalasingam T. Fu H. Bonacina F. Wang G. Morales V. (2022). Loss of voltage-gated hydrogen channel 1 expression reveals heterogeneous metabolic adaptation to intracellular acidification by T cells. JCI Insight 7, e147814–e147819. 10.1172/jci.insight.147814 35472029 PMC9220931

[B32] Corzo C. A. Cotter M. J. Cheng P. Cheng F. Kusmartsev S. Sotomayor E. (2009). Mechanism regulating reactive oxygen species in tumor-induced myeloid-derived suppressor cells. J. Immunol. 182, 5693–5701. 10.4049/jimmunol.0900092 19380816 PMC2833019

[B33] de Grotthuss C. J. T. (2006). Memoir on the decomposition of water and of the bodies that it holds in solution by means of galvanic electricity. Biochim. Biophys. Acta - Bioenerg. 1757, 871–875. 10.1016/j.bbabio.2006.07.004 16962993

[B34] De-la-Rosa V. Suárez-Delgado E. Rangel-Yescas G. E. Islas L. D. (2016). Currents through Hv1 channels deplete protons in their vicinity. J. Gen. Physiol. 147, 127–136. 10.1085/jgp.201511496 26809792 PMC4727945

[B35] DeCoursey T. E. Cherny V. V. DeCoursey A. G. Xu W. Thomas L. L. (2001). Interactions between NADPH oxidase-related proton and electron currents in human eosinophils. J. Physiol. 535, 767–781. 10.1111/j.1469-7793.2001.00767.x 11559774 PMC2278831

[B36] DeCoursey T. E. Cherny V. V. Zhou W. Thomas L. L. (2000). Simultaneous activation of NADPH oxidase-related proton and electron currents in human neutrophils. Proc. Natl. Acad. Sci. U. S. A. 97, 6885–6889. 10.1073/pnas.100047297 10823889 PMC18770

[B37] DeCoursey T. E. (1991). Hydrogen ion currents in rat alveolar epithelial cells. Biophys. J. 60, 1243–1253. 10.1016/S0006-3495(91)82158-0 1722118 PMC1260178

[B38] Decoursey T. E. (2003). Voltage-gated proton channels and other proton transfer pathways. Physiol. Rev. 83, 475–579. 10.1152/physrev.00028.2002 12663866

[B155] DeCoursey T. E. (2018). Voltage and pH sensing by the voltage-gated proton channel, HV1. J. R. Soc. Interface 15, 20180108. 10.1098/rsif.2018.0108 29643227 PMC5938591

[B39] Denko N. C. (2008). Metabolism in the solid tumour. Nat. Rev. Cancer 8, 705–713. Available at: http://www.ncbi.nlm.nih.gov/entrez/query.fcgi?cmd=Retrieve&db=PubMed&dopt=Citation&list_uids=19143055 . 19143055 10.1038/nrc2468

[B40] Dudev T. Musset B. Morgan D. Cherny V. V. Smith S. M. E. Mazmanian K. (2015). Selectivity mechanism of the voltage-gated proton channel, HV1. Sci. Rep. 5, 10320. 10.1038/srep10320 25955978 PMC4429351

[B41] Dunn G. P. Bruce A. T. Ikeda H. Old L. J. Schreiber R. D. (2002). Cancer immunoediting: From immunosurveillance to tumor escape. Nat. Immunol. 3, 991–998. 10.1038/ni1102-991 12407406

[B42] Eder C. DeCoursey T. E. (2001). Voltage-gated proton channels in microglia. Prog. Neurobiol. 64, 277–305. 10.1016/s0301-0082(00)00062-9 11240310

[B43] Egeblad M. Rasch M. G. Weaver V. M. (2010). Dynamic interplay between the collagen scaffold and tumor evolution. Curr. Opin. Cell Biol. 22, 697–706. 10.1016/j.ceb.2010.08.015 20822891 PMC2948601

[B44] Esfahani K. Roudaia L. Buhlaiga N. Del Rincon S. V. Papneja N. Miller W. H. (2020). A review of cancer immunotherapy: From the past, to the present, to the future. Curr. Oncol. 27, S87–S97. 10.3747/co.27.5223 32368178 PMC7194005

[B45] Fisher R. Pusztai L. Swanton C. (2013). Cancer heterogeneity: Implications for targeted therapeutics. Br. J. Cancer 108, 479–485. 10.1038/bjc.2012.581 23299535 PMC3593543

[B46] Fujiwara Y. Kurokawa T. Takeshita K. Kobayashi M. Okochi Y. Nakagawa A. (2012). The cytoplasmic coiled-coil mediates cooperative gating temperature sensitivity in the voltage-gated H+ channel Hv1. Nat. Commun. 3, 816. 10.1038/ncomms1823 22569364

[B47] Gabrilovich D. I. Ostrand-Rosenberg S. Bronte V. (2012). Coordinated regulation of myeloid cells by tumours. Nat. Rev. Immunol. 12, 253–268. 10.1038/nri3175 22437938 PMC3587148

[B48] Gialeli C. Theocharis A. D. Karamanos N. K. (2011). Roles of matrix metalloproteinases in cancer progression and their pharmacological targeting. FEBS J. 278, 16–27. 10.1111/j.1742-4658.2010.07919.x 21087457

[B49] Gianti E. Delemotte L. Klein M. L. Carnevale V. (2016). On the role of water density fluctuations in the inhibition of a proton channel. Proc. Natl. Acad. Sci. U. S. A. 113, E8359–E8368. 10.1073/pnas.1609964114 27956641 PMC5206518

[B50] Glunde K. Guggino S. E. Solaiyappan M. Pathak A. P. Ichikawa Y. Bhujwalla Z. M. (2003). Extracellular acidification alters lysosomal trafficking in human breast cancer cells. Neoplasia 5, 533–545. 10.1016/s1476-5586(03)80037-4 14965446 PMC1502575

[B51] Goel S. Duda D. G. Xu L. Munn L. L. Boucher Y. Fukumura D. (2011). Normalization of the vasculature for treatment of cancer and other diseases. Physiol. Rev. 91, 1071–1121. 10.1152/physrev.00038.2010 21742796 PMC3258432

[B52] Goldin A. L. (2003). Mechanisms of sodium channel inactivation. Curr. Opin. Neurobiol. 13, 284–290. 10.1016/S0959-4388(03)00065-5 12850212

[B53] Gonzalez C. Koch H. P. Drum B. M. Larsson H. P. (2010). Strong cooperativity between subunits in voltage-gated proton channels. Nat. Struct. Mol. Biol. 17, 51–56. 10.1038/nsmb.1739 20023639 PMC2935852

[B54] Gonzalez C. Rebolledo S. Perez M. E. Larsson H. P. (2013). Molecular mechanism of voltage sensing in voltage-gated proton channels. J. Gen. Physiol. 141, 275–285. 10.1085/jgp.201210857 23401575 PMC3581690

[B55] Gordienko D. V. Tare M. Parveen S. Fenech C. J. Robinson C. Bolton T. B. (1996). Voltage-activated proton current in eosinophils from human blood. J. Physiol. 496, 299–316. 10.1113/jphysiol.1996.sp021686 8910217 PMC1160878

[B56] Hamamah S. Gatti J. L. (1998). Role of the ionic environment and internal pH on sperm activity. Hum. Reprod. 13, 20–30. 10.1093/humrep/13.suppl_4.20 10091055

[B57] Hanahan D. Weinberg R. A. (2011). Hallmarks of cancer: The next generation. Cell 144, 646–674. 10.1016/j.cell.2011.02.013 21376230

[B58] Hanahan D. Weinberg R. A. (2000). The hallmarks of cancer. Cell 100, 57–70. 10.1016/S0092-8674(00)81683-9 10647931

[B59] Hassanpour S. H. Dehghani M. (2017). Review of cancer from perspective of molecular. J. Cancer Res. Pract. 4, 127–129. 10.1016/j.jcrpr.2017.07.001

[B60] Helmlinger G. Sckell A. Dellian M. Forbes N. S. Jain R. K. (2002). Acid production in glycolysis-impaired tumors provides new insights into tumor metabolism. Clin. Cancer Res. 8, 1284–1291. Available at: http://www.ncbi.nlm.nih.gov/pubmed/11948144 . 11948144

[B61] Helmlinger G. Yuan F. Dellian M. Jain R. K. (1997). Interstitial pH and pO2 gradients in solid tumors *in vivo*: High-resolution measurements reveal a lack of correlation. Nat. Med. 3, 177–182. 10.1038/nm0297-177 9018236

[B62] Henderson L. M. Chappell J. B. Jones O. T. G. (1988). Internal pH changes associated with the activity of NADPH oxidase of human neutrophils. Further evidence for the presence of an H+ conducting channel. Biochem. J. 251, 563–567. 10.1042/bj2510563 2456757 PMC1149038

[B63] Hille B. (2001). Ion channels of excitable membranes 3rd ed. Sunderland (Mass: Sinauer Associates.

[B64] Hondares E. Brown M. A. Musset B. Morgan D. Cherny V. V. Taubert C. (2014). Enhanced activation of an amino-terminally truncated isoform of the voltage-gated proton channel HVCN1 enriched in malignant B cells. Proc. Natl. Acad. Sci. 111, 18078–18083. 10.1073/pnas.1411390111 25425665 PMC4273330

[B65] Hong L. Kim I. H. Tombola F. (2014). Molecular determinants of Hv1 proton channel inhibition by guanidine derivatives. Proc. Natl. Acad. Sci. 111, 9971–9976. 10.1073/pnas.1324012111 24912149 PMC4103315

[B66] Hong L. Pathak M. M. Kim I. H. Ta D. Tombola F. (2013). Voltage-sensing domain of voltage-gated proton channel Hv1 shares mechanism of block with pore domains. Neuron 77, 274–287. 10.1016/j.neuron.2012.11.013 23352164 PMC3559007

[B67] Huang S. Van Arsdall M. Tedjarati S. McCarty M. Wu W. Langley R. (2002). Contributions of stromal metalloproteinase-9 to angiogenesis and growth of human ovarian carcinoma in mice. J. Natl. Cancer Inst. 94, 1134–1142. 10.1093/jnci/94.15.1134 12165638

[B68] Jin M. Z. Jin W. L. (2020). The updated landscape of tumor microenvironment and drug repurposing. Signal Transduct. Target. Ther. 5, 166. 10.1038/s41392-020-00280-x 32843638 PMC7447642

[B69] Joseph C. Alsaleem M. Orah N. Narasimha P. L. Miligy I. M. Kurozumi S. (2020). Elevated MMP9 expression in breast cancer is a predictor of shorter patient survival. Breast Cancer Res. Treat. 182, 267–282. 10.1007/s10549-020-05670-x 32445177 PMC7297818

[B70] Kai F. B. Drain A. P. Weaver V. M. (2019). The extracellular matrix modulates the metastatic journey. Dev. Cell 49, 332–346. 10.1016/j.devcel.2019.03.026 31063753 PMC6527347

[B71] Kawai T. Tatsumi S. Kihara S. Sakimura K. Okamura Y. (2018). Mechanistic insight into the suppression of microglial ROS production by voltage-gated proton channels (VSOP/Hv1). Channels 12, 1–8. 10.1080/19336950.2017.1385684 28961043 PMC5972804

[B72] Kawanabe A. Okamura Y. (2016). Effects of unsaturated fatty acids on the kinetics of voltage-gated proton channels heterologously expressed in cultured cells. J. Physiol. 594, 595–610. 10.1113/JP271274 26563684 PMC5341712

[B73] Kessenbrock K. Plaks V. Werb Z. (2010). Matrix metalloproteinases: Regulators of the tumor microenvironment. Cell 141, 52–67. 10.1016/j.cell.2010.03.015 20371345 PMC2862057

[B74] Khosrowabadi E. Rivinoja A. Risteli M. Tuomisto A. Salo T. Mäkinen M. J. (2021). SLC4A2 anion exchanger promotes tumour cell malignancy via enhancing net acid efflux across golgi membranes. Cell. Mol. Life Sci. 78, 6283–6304. 10.1007/s00018-021-03890-y 34279699 PMC8429400

[B75] Koch H. P. Kurokawa T. Okochi Y. Sasaki M. Okamura Y. Larsson H. P. (2008). Multimeric nature of voltage-gated proton channels. Proc. Natl. Acad. Sci. U. S. A. 105, 9111–9116. 10.1073/pnas.0801553105 18583477 PMC2449373

[B76] Kulleperuma K. Smith S. M. E. Morgan D. Musset B. Holyoake J. Chakrabarti N. (2013). Construction and validation of a homology model of the human voltage-gated proton channel hHv1. J. Gen. Physiol. 141, 445–465. 10.1085/jgp.201210856 23530137 PMC3607825

[B77] Kusmartsev S. Gabrilovich D. I. (2006). Role of immature myeloid cells in mechanisms of immune evasion in cancer. Cancer Immunol. Immunother. 55, 237–245. 10.1007/s00262-005-0048-z 16047143 PMC1350971

[B78] Le Floch R. Chiche J. Marchiq I. Naïken T. Ilk K. Murray C. M. (2012). Erratum: CD147 subunit of lactate/H+ symporters MCT1 and hypoxia-inducible MCT4 is critical for energetics and growth of glycolytic tumors. Proc. Natl. Acad. Sci. U. S. A. 109, 20166. 10.1073/pnas.1219161109 PMC3189052 21930917

[B79] Lee S. Y. Letts J. A. MacKinnon R. (2008). Dimeric subunit stoichiometry of the human voltage-dependent proton channel Hv1. Proc. Natl. Acad. Sci. U. S. A. 105, 7692–7695. 10.1073/pnas.0803277105 18509058 PMC2409406

[B80] Li Y. Ritzel R. M. He J. Cao T. Sabirzhanov B. Li H. (2021). The voltage-gated proton channel Hv1 plays a detrimental role in contusion spinal cord injury via extracellular acidosis-mediated neuroinflammation. Brain. Behav. Immun. 91, 267–283. 10.1016/j.bbi.2020.10.005 33039662 PMC7749852

[B81] Lishko P. V. Botchkina I. L. Fedorenko A. Kirichok Y. (2010). Acid extrusion from human spermatozoa is mediated by flagellar voltage-gated proton channel. Cell 140, 327–337. 10.1016/j.cell.2009.12.053 20144758

[B82] Litan A. Langhans S. A. (2015). Cancer as a channelopathy: Ion channels and pumps in tumor development and progression. Front. Cell. Neurosci. 9, 86–11. 10.3389/fncel.2015.00086 25852478 PMC4362317

[B83] Lopez-Charcas O. Poisson L. Benouna O. Lemoine R. Chadet S. Pétereau A. (2022). Voltage-gated sodium channel NaV1.5 controls NHE−1−Dependent invasive properties in colon cancer cells. Cancers (Basel) 15, 46. 10.3390/cancers15010046 36612049 PMC9817685

[B84] Lovannisci D. Illek B. Fischer H. (2010). Function of the HVCN1 proton channel in airway epithelia and a naturally occurring mutation, M91T. J. Gen. Physiol. 136, 35–46. 10.1085/jgp.200910379 20548053 PMC2894549

[B85] Lu T. Gabrilovich D. I. (2012). Molecular pathways: Tumor-infiltrating myeloid cells and reactive oxygen species in regulation of tumor microenvironment. Clin. Cancer Res. 18, 4877–4882. 10.1158/1078-0432.CCR-11-2939 22718858 PMC3445728

[B156] Mahaut-Smith M. P. (1989). The effect of zinc on calcium and hydrogen ion currents in intact snail neurones. J. Exp. Biol. 145, 455–464. 10.1242/jeb.145.1.455 22912993

[B86] Meacham C. E. Morrison S. J. (2013). Tumour heterogeneity and cancer cell plasticity. Nature 501, 328–337. 10.1038/nature12624 24048065 PMC4521623

[B87] Mehner C. Hockla A. Miller E. Ran S. Radisky D. C. Radisky E. S. (2014). Tumor cell-produced matrix metalloproteinase 9 (MMP-9) drives malignant progression and metastasis of basal-like triple negative breast cancer. Oncotarget 5, 2736–2749. 10.18632/oncotarget.1932 24811362 PMC4058041

[B88] Mony L. Berger T. K. Isacoff E. Y. (2015). A specialized molecular motion opens the Hv1 voltage-gated proton channel. Nat. Struct. Mol. Biol. 22, 283–290. 10.1038/nsmb.2978 25730777 PMC4385474

[B89] Mony L. Stroebel D. Isacoff E. Y. (2020). Dimer interaction in the Hv1 proton channel. Proc. Natl. Acad. Sci. U. S. A. 117, 20898–20907. 10.1073/pnas.2010032117 32788354 PMC7456152

[B90] Morgan D. Capasso M. Musset B. Cherny V. V. Ríos E. Dyer M. J. S. (2009). Voltage-gated proton channels maintain pH in human neutrophils during phagocytosis. Proc. Natl. Acad. Sci. U. S. A. 106, 18022–18027. 10.1073/pnas.0905565106 19805063 PMC2764923

[B91] Morgan D. Cherny V. V. Murphy R. Katz B. Z. DeCoursey T. E. (2005). The pH dependence of NADPH oxidase in human eosinophils. J. Physiol. 569, 419–431. 10.1113/jphysiol.2005.094748 16195320 PMC1464255

[B92] Morgan D. Cherny V. V. Finnegan A. Bollinger J. Gelb M. H. Decoursey T. E. (2007). Sustained activation of proton channels and NADPH oxidase in human eosinophils and murine granulocytes requires PKC but not cPLA2 alpha activity. J. Physiol. 579, 327–344. 10.1113/jphysiol.2006.124248 17185330 PMC2075394

[B93] Morgan D. Musset B. Kulleperuma K. Smith S. M. E. Rajan S. Cherny V. V. (2013). Peregrination of the selectivity filter delineates the pore of the human: Voltage-gated proton channel hHv1. J. Gen. Physiol. 142, 625–640. 10.1085/jgp.201311045 24218398 PMC3840923

[B94] Movahedi K. Guilliams M. Van Den Bossche J. Van Den Bergh R. Gysemans C. Beschin A. (2008). Identification of discrete tumor-induced myeloid-derived suppressor cell subpopulations with distinct T cell suppressive activity. Blood 111, 4233–4244. 10.1182/blood-2007-07-099226 18272812

[B95] Musset B. Capasso M. Cherny V. V. Morgan D. Bhamrah M. Dyer M. J. S. (2010). Identification of Thr29 as a critical phosphorylation site that activates the human proton channel Hvcn1 in leukocytes. J. Biol. Chem. 285, 5117–5121. 10.1074/jbc.C109.082727 20037153 PMC2820736

[B96] Musset B. DeCoursey T. (2012). Biophysical properties of the voltage-gated proton channel H V 1. Wiley Interdiscip. Rev. Membr. Transp. Signal. 1, 605–620. 10.1002/wmts.55 23050239 PMC3462886

[B97] Musset B. Morgan D. Cherny V. V. MacGlashan D. W. Thomas L. L. Rios E. (2008). A pH-stabilizing role of voltage-gated proton channels in IgE-mediated activation of human basophils. Proc. Natl. Acad. Sci. 105, 11020–11025. 10.1073/pnas.0800886105 18664579 PMC2504794

[B98] Nagaraj S. Gupta K. Pisarev V. Kinarsky L. Sherman S. Kang L. (2007). Altered recognition of antigen is a mechanism of CD8+T cell tolerance in cancer. Nat. Med. 13, 828–835. 10.1038/nm1609 17603493 PMC2135607

[B99] Nanda A. Romanek R. Curnutte J. T. Grinstein S. (1994). Assessment of the contribution of the cytochrome b moiety of the NADPH oxidase to the transmembrane H+ conductance of leukocytes. J. Biol. Chem. 269, 27280–27285. 10.1016/s0021-9258(18)46981-5 7525551

[B100] Ordway B. Gillies R. J. Damaghi M. (2021). Extracellular acidification induces lysosomal dysregulation. Cells 10, 1188–1210. 10.3390/cells10051188 34067971 PMC8152284

[B101] Pang H. Li J. Wang Y. Su X. Gao Y. Li S. J. (2021). Mice lacking the proton channel Hv1 exhibit sex-specific differences in glucose Homeostasis. J. Biol. Chem. 279, 101212. 10.1016/j.jbc.2021.101212 PMC8503595 34547291

[B102] Pang H. Wang X. Zhao S. Xi W. Lv J. Qin J. (2020). Loss of the voltage-gated proton channel Hv1 decreases insulin secretion and leads to hyperglycemia and glucose intolerance in mice. J. Biol. Chem. 295, 3601–3613. 10.1074/jbc.RA119.010489 31949049 PMC7076216

[B103] Panyi G. Beeton C. Felipe A. (2014). Ion channels and anti-cancer immunity. Philos. Trans. R. Soc. B Biol. Sci. 369, 20130106. 10.1098/rstb.2013.0106 PMC3917360 24493754

[B104] Peng J. Yi M.-H. Jeong H. McEwan P. P. Zheng J. Wu G. (2021). The voltage-gated proton channel Hv1 promotes microglia-astrocyte communication and neuropathic pain after peripheral nerve injury. Mol. Brain 14, 99. 10.1186/s13041-021-00812-8 34183051 PMC8240390

[B105] Peranzoni E. Zilio S. Marigo I. Dolcetti L. Zanovello P. Mandruzzato S. (2010). Myeloid-derived suppressor cell heterogeneity and subset definition. Curr. Opin. Immunol. 22, 238–244. 10.1016/j.coi.2010.01.021 20171075

[B106] Pértega-Gomes N. Vizcaíno J. R. Miranda-Gonçalves V. Pinheiro C. Silva J. Pereira H. (2011). Monocarboxylate transporter 4 (MCT4) and CD147 overexpression is associated with poor prognosis in prostate cancer. BMC Cancer 11, 312. 10.1186/1471-2407-11-312 21787388 PMC3157459

[B107] Qiu F. Chamberlin A. Watkins B. M. Ionescu A. Perez M. E. Barro-Soria R. (2016). Molecular mechanism of Zn2+ inhibition of a voltage-gated proton channel. Proc. Natl. Acad. Sci. U. S. A. 113, E5962–E5971. 10.1073/pnas.1604082113 27647906 PMC5056077

[B108] Qiu F. Rebolledo S. Gonzalez C. Larsson H. P. (2013). Subunit interactions during cooperative opening of voltage-gated proton channels. Neuron 77, 288–298. 10.1016/j.neuron.2012.12.021 23352165 PMC3558936

[B109] Qiu G. Z. Jin M. Z. Dai J. X. Sun W. Feng J. H. Jin W. L. (2017). Reprogramming of the tumor in the hypoxic niche: The emerging concept and associated therapeutic strategies. Trends Pharmacol. Sci. 38, 669–686. 10.1016/j.tips.2017.05.002 28602395

[B110] RamaKrishnan A. M. Sankaranarayanan K. (2016). Understanding autoimmunity: The ion channel perspective. Autoimmun. Rev. 15, 585–620. 10.1016/j.autrev.2016.02.004 26854401

[B111] Ramsey I. S. Mokrab Y. Carvacho I. Sands Z. A. Sansom M. S. P. Clapham D. E. (2010). An aqueous H+ permeation pathway in the voltage-gated proton channel Hv1. Nat. Struct. Mol. Biol. 17, 869–875. 10.1038/nsmb.1826 20543828 PMC4035905

[B112] Ramsey I. S. Moran M. M. Chong J. A. Clapham D. E. (2006). A voltage-gated proton-selective channel lacking the pore domain. Nature 440, 1213–1216. 10.1038/nature04700 16554753 PMC4084761

[B113] Ribeiro-Silva L. Queiroz F. O. Da Silva A. M. B. Hirata A. E. Arcisio-Miranda M. (2016). Voltage-gated proton channel in human glioblastoma multiforme cells. ACS Chem. Neurosci. 7, 864–869. 10.1021/acschemneuro.6b00083 27225904

[B114] Rosenkranz E. Metz C. H. D. Maywald M. Hilgers R. D. Weßels I. Senff T. (2016). Zinc supplementation induces regulatory T cells by inhibition of Sirt-1 deacetylase in mixed lymphocyte cultures. Mol. Nutr. Food Res. 60, 661–671. 10.1002/mnfr.201500524 26614004

[B115] Sakata S. Kurokawa T. Nørholm M. H. H. H. Takagi M. Okochi Y. von Heijne G. (2010). Functionality of the voltage-gated proton channel truncated in S4. Proc. Natl. Acad. Sci. 107, 2313–2318. 10.1073/pnas.0911868107 20018719 PMC2836681

[B116] Sasaki M. Takagi M. Okamura Y. (2006). A voltage sensor-domain protein is a voltage-gated proton channel. Science 312, 589–592. 10.1126/science.1122352 16556803

[B117] Sasaki M. Tojo A. Okochi Y. Miyawaki N. Kamimura D. Yamaguchi A. (2013). Autoimmune disorder phenotypes in *Hvcn1* -deficient mice. Biochem. J. 450, 295–301. 10.1042/BJ20121188 23231444

[B118] Schilling T. Gratopp A. DeCoursey T. E. Eder C. (2002). Voltage-activated proton currents in human lymphocytes. J. Physiol. 545, 93–105. 10.1113/jphysiol.2002.028878 12433952 PMC2290658

[B119] Schladt T. M. Berger T. K. (2020). Voltage and pH difference across the membrane control the S4 voltage-sensor motion of the Hv1 proton channel. Sci. Rep. 10, 21293–21313. 10.1038/s41598-020-77986-z 33277511 PMC7718894

[B120] Sennoune S. R. Bakunts K. Martínez G. M. Chua-Tuan J. L. Kebir Y. Attaya M. N. (2004). Vacuolar H+-ATPase in human breast cancer cells with distinct metastatic potential: Distribution and functional activity. Am. J. Physiol. - Cell Physiol. 286, 1443–1452. 10.1152/ajpcell.00407.2003 14761893

[B121] Serafini P. (2013). Myeloid derived suppressor cells in physiological and pathological conditions: The good, the bad, and the ugly. Immunol. Res. 57, 172–184. 10.1007/s12026-013-8455-2 24203443

[B122] Shin H. Kim J. Song J. H. (2015). Clozapine and olanzapine inhibit proton currents in BV2 microglial cells. Eur. J. Pharmacol. 755, 74–79. 10.1016/j.ejphar.2015.03.003 25771455

[B123] Shin H. Song J. H. (2014). Antipsychotics, chlorpromazine and haloperidol inhibit voltage-gated proton currents in BV2 microglial cells. Eur. J. Pharmacol. 738, 256–262. 10.1016/j.ejphar.2014.05.049 24990667

[B124] Song J. H. Marszalec W. Kai L. Yeh J. Z. Narahashi T. (2012). Antidepressants inhibit proton currents and tumor necrosis factor-α production in BV2 microglial cells. Brain Res. 1435, 15–23. 10.1016/j.brainres.2011.11.041 22177663

[B125] Stagg J. Johnstone R. W. Smyth M. J. (2007). From cancer immunosurveillance to cancer immunotherapy. Immunol. Rev. 220, 82–101. 10.1111/j.1600-065X.2007.00566.x 17979841

[B126] Takeshita K. Sakata S. Yamashita E. Fujiwara Y. Kawanabe A. Kurokawa T. (2014). X-ray crystal structure of voltage-gated proton channel. Nat. Struct. Mol. Biol. 21, 352–357. 10.1038/nsmb.2783 24584463

[B127] Tang D. Yang Y. Xiao Z. Xu J. Yang Q. Dai H. (2020). Scorpion toxin inhibits the voltage-gated proton channel using a Zn2+-like long-range conformational coupling mechanism. Br. J. Pharmacol. 177, 2351–2364. 10.1111/bph.14984 31975366 PMC7174885

[B128] Tesi R. J. (2019). MDSC; the most important cell you have never heard of. Trends Pharmacol. Sci. 40, 4–7. 10.1016/j.tips.2018.10.008 30527590

[B129] Thistlethwaite A. J. Leeper D. B. Moylan D. J. Nerlinger R. E. (1985). pH distribution in human tumors. Int. J. Radiat. Oncol. 11, 1647–1652. 10.1016/0360-3016(85)90217-2 4030433

[B130] Thomas R. C. Meech R. W. (1982). Hydrogen ion currents and intracellular pH in depolarized voltage-clamped snail neurones. Nature 299, 826–828. 10.1038/299826a0 7133121

[B131] Tombola F. Ulbrich M. H. Isacoff E. Y. (2008). The voltage-gated proton channel Hv1 has two pores, each controlled by one voltage sensor. Neuron 58, 546–556. 10.1016/j.neuron.2008.03.026 18498736 PMC2430592

[B132] Tombola F. Ulbrich M. H. Kohout S. C. Isacoff E. Y. (2010). The opening of the two pores of the Hv1 voltage-gated proton channel is tuned by cooperativity. Nat. Struct. Mol. Biol. 17, 44–50. 10.1038/nsmb.1738 20023640 PMC2925041

[B133] Umansky V. Blattner C. Gebhardt C. Utikal J. (2016). The role of myeloid-derived suppressor cells (MDSC) in cancer progression. Vaccines 4. 10.3390/vaccines4040036 PMC5192356 27827871

[B134] Van Wart H. E. Birkedal-Hansen H. (1990). The cysteine switch: A principle of regulation of metalloproteinase activity with potential applicability to the entire matrix metalloproteinase gene family. Proc. Natl. Acad. Sci. U. S. A. 87, 5578–5582. 10.1073/pnas.87.14.5578 2164689 PMC54368

[B135] Ventura C. Leon I. E. Asuaje A. Martín P. Enrique N. Núñez M. (2020). Differential expression of the long and truncated Hv1 isoforms in breast-cancer cells. J. Cell. Physiol. 235, 8757–8767. 10.1002/jcp.29719 32324259

[B136] Vihinen P. Ala-aho R. Kahari V.-M. (2005). Matrix metalloproteinases as therapeutic targets in cancer. Curr. Cancer Drug Targets 5, 203–220. 10.2174/1568009053765799 15892620

[B137] Wang F. Ma X.-R. Wu Y. Xu Y.-C. Gu H.-M. Wang D.-X. (2021). Neutralization of Hv1/HVCN1 with antibody enhances microglia/macrophages myelin clearance by promoting their migration in the brain. Front. Cell. Neurosci. 15, 768059–768110. 10.3389/fncel.2021.768059 34744634 PMC8570284

[B138] Wang Y. Li S. J. Pan J. Che Y. Yin J. Zhao Q. (2011). Specific expression of the human voltage-gated proton channel Hv1 in highly metastatic breast cancer cells, promotes tumor progression and metastasis. Biochem. Biophys. Res. Commun. 412, 353–359. 10.1016/j.bbrc.2011.07.102 21821008

[B139] Wang Y. Li S. J. Wu X. Che Y. Li Q. (2012). Clinicopathological and biological significance of human voltage-gated proton channel Hv1 protein overexpression in breast cancer. J. Biol. Chem. 287, 13877–13888. 10.1074/jbc.M112.345280 22367212 PMC3340163

[B140] Wang Y. Wu X. Li Q. Zhang S. Li S. J. (2013a). Human voltage-gated proton channel Hv1: A new potential biomarker for diagnosis and prognosis of colorectal cancer. PLoS One 8, e70550. 10.1371/journal.pone.0070550 23940591 PMC3734282

[B141] Wang Y. Zhang S. Li S. J. (2013b). Zn2+ induces apoptosis in human highly metastatic SHG-44 glioma cells, through inhibiting activity of the voltage-gated proton channel Hv1. Biochem. Biophys. Res. Commun. 438, 312–317. 10.1016/j.bbrc.2013.07.067 23891691

[B142] Webb B. A. Chimenti M. Jacobson M. P. Barber D. L. (2011). Dysregulated pH: A perfect storm for cancer progression. Nat. Rev. Cancer 11, 671–677. 10.1038/nrc3110 21833026

[B143] Winkler J. Abisoye-Ogunniyan A. Metcalf K. J. Werb Z. (2020). Concepts of extracellular matrix remodelling in tumour progression and metastasis. Nat. Commun. 11, 5120–5219. 10.1038/s41467-020-18794-x 33037194 PMC7547708

[B144] Yang L. DeBusk L. M. Fukuda K. Fingleton B. Green-Jarvis B. Shyr Y. (2004). Expansion of myeloid immune suppressor Gr+CD11b+ cells in tumor-bearing host directly promotes tumor angiogenesis. Cancer Cell 6, 409–421. 10.1016/j.ccr.2004.08.031 15488763

[B145] Yang Y. (2015). Cancer immunotherapy: Harnessing the immune system to battle cancer. J. Clin. Invest. 125, 3335–3337. 10.1172/JCI83871 26325031 PMC4588312

[B146] Youn J.-I. Nagaraj S. Collazo M. Gabrilovich D. I. (2008). Subsets of myeloid-derived suppressor cells in tumor-bearing mice. J. Immunol. 181, 5791–5802. 10.4049/jimmunol.181.8.5791 18832739 PMC2575748

[B147] Yousef E. M. Tahir M. R. St-Pierre Y. Gaboury L. A. (2014). MMP-9 expression varies according to molecular subtypes of breast cancer. BMC Cancer 14, 609–612. 10.1186/1471-2407-14-609 25151367 PMC4150970

[B148] Yu M. Lee W. W. Tomar D. Pryshchep S. Czesnikiewicz-Guzik M. Lamar D. L. (2011). Regulation of T cell receptor signaling by activation-induced zinc influx. J. Exp. Med. 208, 775–785. 10.1084/jem.20100031 21422171 PMC3135340

[B149] Yu T. Lu Q. Ou X. Cao D. Yu Q. (2014). Association of sedentary behavior with the expression levels of biomarkers in colorectal cancer: Clinical analysis of 228 patients. Tohoku J. Exp. Med. 232, 167–176. 10.1620/tjem.232.167 24621823

[B150] Zhang Q. Ren Y. Mo Y. Guo P. Liao P. Luo Y. (2022). Inhibiting Hv1 channel in peripheral sensory neurons attenuates chronic inflammatory pain and opioid side effects. Cell Res. 32, 461–476. 10.1038/s41422-022-00616-y 35115667 PMC9061814

[B151] Zhao C. Hong L. Galpin J. D. Riahi S. Lim V. T. Webster P. D. (2021). HIFs: New arginine mimic inhibitors of the hv1 channel with improved VSD–ligand interactions. J. Gen. Physiol. 153, e202012832. 10.1085/jgp.202012832 34228044 PMC8263924

[B152] Zhao Q. Che Y. Li Q. Zhang S. Gao Y. T. Wang Y. (2015). The voltage-gated proton channel Hv1 is expressed in pancreatic islet β-cells and regulates insulin secretion. Biochem. Biophys. Res. Commun. 468, 746–751. 10.1016/j.bbrc.2015.11.027 26559003

[B153] Zhao R. Kennedy K. De Blas G. A. Orta G. Pavarotti M. A. Arias R. J. (2018). Role of human Hv1 channels in sperm capacitation and white blood cell respiratory burst established by a designed peptide inhibitor. Proc. Natl. Acad. Sci. U. S. A. 115, E11847–E11856. 10.1073/pnas.1816189115 30478045 PMC6294887

[B154] Zhu X. Mose E. Zimmermann N. (2013). Proton channel HVCN1 is required for effector functions of mouse eosinophils. BMC Immunol. 14, 24. 10.1186/1471-2172-14-24 23705768 PMC3668235

